# Supplementary material for: Seasonal Variation of Glucosinolate Hydrolysis Products in Commercial White and Red Cabbages (Brassica oleracea var. capitata)
Source: Foods. 2020 Nov 17;9(11):1682. doi: 10.3390/foods9111682 (PMC7698573; doi:10.3390/foods9111682)
Supplement: Supplementary file 1 [file Table S1.pdf › Table S1.pdf]

Supplier	Date of purchase	Cabbage variety	Replicate	Grower	Origin of cultivation	cabbage type	soil type	fertilization	Certification mark	Size and shape	Description	weight [kg]	Harvest/season	ng term stored cabbag	Storage conditions before food retailer	Storage at food retailer	Storage conditions at food retailer

Table S1: Overview of white and red cabbages purchased from three different food retailers or harvested freshly from the field (Großbeeren, D). The origin and cultivation background of commercial cabbages was investigated by interrogating service staff at supermarkets and by contacting growers.

Supplier	Sample	Date of purchase	Cabbage variety	Replicate	Grower	Origin of cultivation	cabbage type	soil type	fertilization	Certification mark	Size and shape	Description	weight [kg]	Harvest/season	ng term stored cabbag	Lagerungsdauer vor dem Verkauf	Storage at food retailer	Storage conditions at food retailer
CON$_1$	S1	4-Sep-2019	red cabbage	1	Conventional grower No. 1	DE, 25792, Neuenkirchen	early cabbage	marsh soil	Urea, calcium ammonium nitrate, phosphate and potash	QS-Siegel	small, round	loose leaves, not glossy	no data	August	no	1-2 days 6-8°C, 1-2 days 6°C, then center of distribution	2 days	day and night in vegetable shelf (20 °C with air conditioning)
CON$_1$	S1	4-Sep-2019	red cabbage	2	Conventional grower No. 1	DE, 25792, Neuenkirchen	early cabbage	marsh soil	Urea, calcium ammonium nitrate, phosphate and potash	QS-Siegel	small, round	loose leaves, not glossy	no data	August	no	1-2 days 6-8°C, 1-2 days 6°C, then center of distribution	2 days	day and night in vegetable shelf (20 °C with air conditioning)
CON$_1$	S1	4-Sep-2019	red cabbage	3	Conventional grower No. 1	DE, 25792, Neuenkirchen	early cabbage	marsh soil	Urea, calcium ammonium nitrate, phosphate and potash	QS-Siegel	small, round	loose leaves, not glossy	no data	August	no	1-2 days 6-8°C, 1-2 days 6°C, then center of distribution	2 days	day and night in vegetable shelf (20 °C with air conditioning)
CON$_1$	S1	4-Sep-2019	white cabbage	1	Conventional grower No. 1	DE, 25792, Neuenkirchen	early cabbage	marsh soil	Urea, calcium ammonium nitrate, phosphate and potash	QS-Siegel	big, round	loose leaves, glossy	no data	August	no	1-2 days 6-8°C, 1-2 days 6°C, then center of distribution	2 days	day and night in vegetable shelf (20 °C with air conditioning)
CON$_1$	S1	4-Sep-2019	white cabbage	2	Conventional grower No. 1	DE, 25792, Neuenkirchen	early cabbage	marsh soil	Urea, calcium ammonium nitrate, phosphate and potash	QS-Siegel	big, round	loose leaves, glossy, with feeding sites	no data	August	no	1-2 days 6-8°C, 1-2 days 6°C, then center of distribution	2 days	day and night in vegetable shelf (20 °C with air conditioning)
CON$_1$	S1	4-Sep-2019	white cabbage	3	Conventional grower No. 1	DE, 25792, Neuenkirchen	early cabbage	marsh soil	Urea, calcium ammonium nitrate, phosphate and potash	QS-Siegel	big, round	loose leaves, glossy, with hole, damaged	no data	August	no	1-2 days 6-8°C, 1-2 days 6°C, then center of distribution	2 days	day and night in vegetable shelf (20 °C with air conditioning)
CON$_2$	S1	5-Sep-2019	red cabbage	1	Conventional grower No. 1	DE, 25792, Neuenkirchen	early cabbage	marsh soil	Urea, calcium ammonium nitrate, phosphate and potash	QS-Siegel	medium-size, round	tight leaves	no data	August	no	1-2 days 6-8°C, then center of distribution	2 days	7-10 °C day/night in cooling counter
CON$_2$	S1	5-Sep-2019	red cabbage	2	Conventional grower No. 1	DE, 25792, Neuenkirchen	early cabbage	marsh soil	Urea, calcium ammonium nitrate, phosphate and potash	QS-Siegel	big, round	damaged	no data	August	no	1-2 days 6-8°C, then center of distribution	2 days	7-10 °C day/night in cooling counter
CON$_2$	S1	5-Sep-2019	red cabbage	3	Conventional grower No. 1	DE, 25792, Neuenkirchen	early cabbage	marsh soil	Urea, calcium ammonium nitrate, phosphate and potash	QS-Siegel	medium-size, round	old, limp leaves	no data	August	no	1-2 days 6-8°C, then center of distribution	2 days	7-10 °C day/night in cooling counter
CON$_2$	S1	5-Sep-2019	white cabbage	1	Conventional grower No. 1	DE, 25792, Neuenkirchen	early cabbage	marsh soil	Urea, calcium ammonium nitrate, phosphate and potash	QS-Siegel	big	loose leaves, damaged	no data	August	no	1-2 days 6-8°C, then center of distribution	1 day	7-10 °C day/night in cooling counter
CON$_2$	S1	5-Sep-2019	white cabbage	2	Conventional grower No. 1	DE, 25792, Neuenkirchen	early cabbage	marsh soil	Urea, calcium ammonium nitrate, phosphate and potash	QS-Siegel	big	glossy leaves	no data	August	no	1-2 days 6-8°C, then center of distribution	1 day	7-10 °C day/night in cooling counter
CON$_2$	S1	5-Sep-2019	white cabbage	3	Conventional grower No. 1	DE, 25792, Neuenkirchen	early cabbage	marsh soil	Urea, calcium ammonium nitrate, phosphate and potash	QS-Siegel	big	older leaves	no data	August	no	1-2 days 6-8°C, then center of distribution	1 day	7-10 °C day/night in cooling counter
ORG$_1$	S1	9-Sep-2019	red cabbage	1	Organic grower No. 1	DE, 17237, Blankensee (Mecklenburg)	autumn cabbage, hybrid cultivar	loamy sandy soil	200kg/ha hair-meal pellets	Bioland, EU organic label	big, round	glossy, fresh	2.25	September	no	1-2 days 2-6 °C , then 1-3 days 4-7°C, then within 48 h to food retailer	0 days	10 °C day/night in cooling counter
ORG$_1$	S1	9-Sep-2019	red cabbage	2	Organic grower No. 1	DE, 17237, Blankensee (Mecklenburg)	autumn cabbage, hybrid cultivar	loamy sandy soil	200kg/ha hair-meal pellets	Bioland, EU organic label	big, round	tight leaves, one damaged site (white looking)	1.97	September	no	1-2 days 2-6 °C , then 1-3 days 4-7°C, then within 48 h to food retailer	0 days	10 °C day/night in cooling counter
ORG$_1$	S1	9-Sep-2019	red cabbage	3	Organic grower No. 1	DE, 17237, Blankensee (Mecklenburg)	autumn cabbage, hybrid cultivar	loamy sandy soil	200kg/ha hair-meal pellets	Bioland, EU organic label	small, oval	tight leaves	1.23	September	no	1-2 days 2-6 °C , then 1-3 days 4-7°C, then within 48 h to food retailer	0 days	10 °C day/night in cooling counter
ORG$_1$	S1	9-Sep-2019	white cabbage	1	Organic grower No. 1	DE, 17237, Blankensee (Mecklenburg)	autumn cabbage, hybrid cultivar	loamy sandy soil	200kg/ha hair-meal pellets	Bioland, EU organic label	medium-size, round	limp leaves, brown sites, feeding sites	1.45	September	no	1-2 days 2-6 °C , then 1-3 days 4-7°C, then within 48 h to food retailer	0 days	10 °C day/night in cooling counter
ORG$_1$	S1	9-Sep-2019	white cabbage	2	Organic grower No. 1	DE, 17237, Blankensee (Mecklenburg)	autumn cabbage, hybrid cultivar	loamy sandy soil	200kg/ha hair-meal pellets	Bioland, EU organic label	medium-size, round	soft, brown site	1.27	September	no	1-2 days 2-6 °C , then 1-3 days 4-7°C, then within 48 h to food retailer	0 days	10 °C day/night in cooling counter
ORG$_1$	S1	9-Sep-2019	white cabbage	3	Organic grower No. 1	DE, 17237, Blankensee (Mecklenburg)	autumn cabbage, hybrid cultivar	loamy sandy soil	200kg/ha hair-meal pellets	Bioland, EU organic label	medium-size, round	with a cut	1.24	September	no	1-2 days 2-6 °C , then 1-3 days 4-7°C, then within 48 h to food retailer	0 days	10 °C day/night in cooling counter
CON$_1$	S2	16-Sep-2019	red cabbage	1	Conventional grower No. 1	DE, 25792, Neuenkirchen	autumn cabbage	marsh soil	Urea, calcium ammonium nitrate, phosphate and potash	QS-Siegel	big, round	curled leaves	3.20	September	no	1-2 days 6°C, then center of distribution (1-2 days)	3 days	day and night in vegetable shelf (20 °C with air conditioning)
CON$_1$	S2	16-Sep-2019	red cabbage	2	Conventional grower No. 1	DE, 25792, Neuenkirchen	autumn cabbage	marsh soil	Urea, calcium ammonium nitrate, phosphate and potash	QS-Siegel	small, oval	wrinkled	1.62	September	no	1-2 days 6°C, then center of distribution (1-2 days)	3 days	day and night in vegetable shelf (20 °C with air conditioning)
CON$_1$	S2	16-Sep-2019	red cabbage	3	Conventional grower No. 1	DE, 25792, Neuenkirchen	autumn cabbage	marsh soil	Urea, calcium ammonium nitrate, phosphate and potash	QS-Siegel	medium-size, round	tight leaves	2.23	September	no	1-2 days 6°C, then center of distribution (1-2 days)	3 days	day and night in vegetable shelf (20 °C with air conditioning)
CON$_1$	S2	16-Sep-2019	white cabbage	1	Conventional grower No. 1	DE, 25792, Neuenkirchen	autumn cabbage	marsh soil	Urea, calcium ammonium nitrate, phosphate and potash	QS-Siegel	big, round	slightly damaged	2.87	September	no	1-2 days 6°C, then center of distribution (1-2 days)	2 days	day and night in vegetable shelf (20 °C with air conditioning)
CON$_1$	S2	16-Sep-2019	white cabbage	2	Conventional grower No. 1	DE, 25792, Neuenkirchen	autumn cabbage	marsh soil	Urea, calcium ammonium nitrate, phosphate and potash	QS-Siegel	medium-size, round	curled leaves, slightly damaged	2.14	September	no	1-2 days 6°C, then center of distribution (1-2 days)	2 days	day and night in vegetable shelf (20 °C with air conditioning)
CON$_1$	S2	16-Sep-2019	white cabbage	3	Conventional grower No. 1	DE, 25792, Neuenkirchen	autumn cabbage	marsh soil	Urea, calcium ammonium nitrate, phosphate and potash	QS-Siegel	medium-size, round	tight leaves	2.35	September	no	1-2 days 6°C, then center of distribution (1-2 days)	2 days	day and night in vegetable shelf (20 °C with air conditioning)
CON$_2$	S2	16-Sep-2019	red cabbage	1	Conventional grower No. 1	DE, 25792, Neuenkirchen	autumn cabbage	marsh soil	Urea, calcium ammonium nitrate, phosphate and potash	QS-Siegel	medium-size, round	loose leaves	1.93	September	no	1-2 days 6-8°C, then center of distribution	4 days	7-10 °C day/night in cooling counter
CON$_2$	S2	16-Sep-2019	red cabbage	2	Conventional grower No. 1	DE, 25792, Neuenkirchen	autumn cabbage	marsh soil	Urea, calcium ammonium nitrate, phosphate and potash	QS-Siegel	small	tight leaves	1.39	September	no	1-2 days 6-8°C, then center of distribution	4 days	7-10 °C day/night in cooling counter
CON$_2$	S2	16-Sep-2019	red cabbage	3	Conventional grower No. 1	DE, 25792, Neuenkirchen	autumn cabbage	marsh soil	Urea, calcium ammonium nitrate, phosphate and potash	QS-Siegel	very small	tight leaves	0.76	September	no	1-2 days 6-8°C, then center of distribution	4 days	7-10 °C day/night in cooling counter
CON$_2$	S2	16-Sep-2019	white cabbage	1	Conventional grower No. 1	DE, 25792, Neuenkirchen	autumn cabbage	marsh soil	Urea, calcium ammonium nitrate, phosphate and potash	QS-Siegel	big, round	tight leaves	2.31	September	no	1-2 days 6-8°C, then center of distribution	4 days	7-10 °C day/night in cooling counter
CON$_2$	S2	16-Sep-2019	white cabbage	2	Conventional grower No. 1	DE, 25792, Neuenkirchen	autumn cabbage	marsh soil	Urea, calcium ammonium nitrate, phosphate and potash	QS-Siegel	medium-size, round	loose, limp, old leaves	1.96	September	no	1-2 days 6-8°C, then center of distribution	4 days	7-10 °C day/night in cooling counter
CON$_2$	S2	16-Sep-2019	white cabbage	3	Conventional grower No. 1	DE, 25792, Neuenkirchen	autumn cabbage	marsh soil	Urea, calcium ammonium nitrate, phosphate and potash	QS-Siegel	big, round	damaged with feeding sites	2.67	September	no	1-2 days 6-8°C, then center of distribution	4 days	7-10 °C day/night in cooling counter
ORG$_1$	S2	19-Sep-2019	red cabbage	1	Organic grower No. 2	DE, 25761, Hedwigenkoog	autumn cabbage, seedfast	sea marsh soil	compost	Demeter, EU organic label	small, oval	minor feeding sites, tight leaves, fresh	0.91	September	no	1-2 days 7-8 °C , then 1-3 days center of distribution, then within 48 h to food retailer	no reliable data, maximum 8 days	10 °C day/night in cooling counter
ORG$_1$	S2	19-Sep-2019	red cabbage	2	Organic grower No. 2	DE, 25761, Hedwigenkoog	autumn cabbage, seedfast	sea marsh soil	compost	Demeter, EU organic label	small, oval	slightly damaged, tight leaves	0.87	September	no	1-2 days 7-8 °C , then 1-3 days center of distribution, then within 48 h to food retailer	no reliable data, maximum 8 days	10 °C day/night in cooling counter
ORG$_1$	S2	19-Sep-2019	red cabbage	3	Organic grower No. 2	DE, 25761, Hedwigenkoog	autumn cabbage, seedfast	sea marsh soil	compost	Demeter, EU organic label	medium-size, oval	soft leaves, undamaged	1.14	September	no	1-2 days 7-8 °C , then 1-3 days center of distribution, then within 48 h to food retailer	no reliable data, maximum 8 days	10 °C day/night in cooling counter
ORG$_1$	S2	19-Sep-2019	white cabbage	1	Organic grower No. 2	DE, 25761, Hedwigenkoog	autumn cabbage, seedfast	sea marsh soil	compost	Demeter, EU organic label	small, round	limp leaves, unfresh, with holes, dirty	0.98	September	no	1-2 days 7-8 °C , then 1-3 days center of distribution, then within 48 h to food retailer	no reliable data, maximum 8 days	10 °C day/night in cooling counter
ORG$_1$	S2	19-Sep-2019	white cabbage	2	Organic grower No. 2	DE, 25761, Hedwigenkoog	autumn cabbage, seedfast	sea marsh soil	compost	Demeter, EU organic label	medium-size, round	limp leaves, dirty, with holes and feeding sites	1.47	September	no	1-2 days 7-8 °C , then 1-3 days center of distribution, then within 48 h to food retailer	no reliable data, maximum 8 days	10 °C day/night in cooling counter
ORG$_1$	S2	19-Sep-2019	white cabbage	3	Organic grower No. 2	DE, 25761, Hedwigenkoog	autumn cabbage, seedfast	sea marsh soil	compost	Demeter, EU organic label	medium-size, round	dirty, tight leaves	1.17	September	no	1-2 days 7-8 °C , then 1-3 days center of distribution, then within 48 h to food retailer	no reliable data, maximum 8 days	10 °C day/night in cooling counter
CON$_1$	S3	30-Sep-2019	red cabbage	1	Conventional grower No. 1	DE, 25792, Neuenkirchen	autumn cabbage	marsh soil	Urea, calcium ammonium nitrate, phosphate and potash	QS-Siegel	big, round	loose leaves, wrinkled, soft core, very limp and old	2.50	September	no	1-2 days 6-8°C, 1-2 days 6°C, then center of distribution	4 days	day and night in vegetable shelf (20 °C with air conditioning)
CON$_1$	S3	30-Sep-2019	red cabbage	2	Conventional grower No. 1	DE, 25792, Neuenkirchen	autumn cabbage	marsh soil	Urea, calcium ammonium nitrate, phosphate and potash	QS-Siegel	small to medium-size, round	limp, old, unfresh	1.94	September	no	1-2 days 6-8°C, 1-2 days 6°C, then center of distribution	4 days	day and night in vegetable shelf (20 °C with air conditioning)
CON$_1$	S3	30-Sep-2019	red cabbage	3	Conventional grower No. 1	DE, 25792, Neuenkirchen	autumn cabbage	marsh soil	Urea, calcium ammonium nitrate, phosphate and potash	QS-Siegel	big, oval	only slightly limp, tighter leaves, with a fresh cut	2.43	September	no	1-2 days 6-8°C, 1-2 days 6°C, then center of distribution	4 days	day and night in vegetable shelf (20 °C with air conditioning)
CON$_1$	S3	30-Sep-2019	white cabbage	1	Conventional grower No. 1	DE, 25792, Neuenkirchen	autumn cabbage	marsh soil	Urea, calcium ammonium nitrate, phosphate and potash	QS-Siegel	big, round	brown sites, dirty, curled leaves	3.16	September	no	1-2 days 6-8°C, 1-2 days 6°C, then center of distribution	4 days	day and night in vegetable shelf (20 °C with air conditioning)
CON$_1$	S3	30-Sep-2019	white cabbage	2	Conventional grower No. 1	DE, 25792, Neuenkirchen	autumn cabbage	marsh soil	Urea, calcium ammonium nitrate, phosphate and potash	QS-Siegel	big, round	fresh	3.14	September	no	1-2 days 6-8°C, 1-2 days 6°C, then center of distribution	4 days	day and night in vegetable shelf (20 °C with air conditioning)
CON$_1$	S3	30-Sep-2019	white cabbage	3	Conventional grower No. 1	DE, 25792, Neuenkirchen	autumn cabbage	marsh soil	Urea, calcium ammonium nitrate, phosphate and potash	QS-Siegel	big, round	brown sites, curled leaves, with feeding sites	2.60	September	no	1-2 days 6-8°C, 1-2 days 6°C, then center of distribution	4 days	day and night in vegetable shelf (20 °C with air conditioning)
CON$_2$	S3	30-Sep-2019	red cabbage	1	Conventional grower No. 1	DE, 25792, Neuenkirchen	autumn cabbage	marsh soil	Urea, calcium ammonium nitrate, phosphate and potash	QS-Siegel	medium-size, oval	limp, bit soft feeding site	n.a.	September	no	1-2 days 6-8°C, then center of distribution	3 days	7-10 °C day/night in cooling counter
CON$_2$	S3	30-Sep-2019	red cabbage	2	Conventional grower No. 1	DE, 25792, Neuenkirchen	autumn cabbage	marsh soil	Urea, calcium ammonium nitrate, phosphate and potash	QS-Siegel	medium-size, oval	curled leaves, old, limp, with a feeding site	2.10	September	no	1-2 days 6-8°C, then center of distribution	3 days	7-10 °C day/night in cooling counter
CON$_2$	S3	1-Oct-2019	red cabbage	3	Conventional grower No. 1	DE, 25792, Neuenkirchen	autumn cabbage	marsh soil	Urea, calcium ammonium nitrate, phosphate and potash	QS-Siegel	medium-size, oval	fresh, with a cut	1.29	September	no	1-2 days 6-8°C, then center of distribution	1 day	7-10 °C day/night in cooling counter
CON$_2$	S3	30-Sep-2019	white cabbage	1	Conventional grower No. 1	DE, 25792, Neuenkirchen	autumn cabbage	marsh soil	Urea, calcium ammonium nitrate, phosphate and potash	QS-Siegel	small, round	very fresh	1.55	September	no	1-2 days 6-8°C, then center of distribution	3 days	7-10 °C day/night in cooling counter
CON$_2$	S3	30-Sep-2019	white cabbage	2	Conventional grower No. 1	DE, 25792, Neuenkirchen	autumn cabbage	marsh soil	Urea, calcium ammonium nitrate, phosphate and potash	QS-Siegel	small, round	very fresh	1.87	September	no	1-2 days 6-8°C, then center of distribution	3 days	7-10 °C day/night in cooling counter
CON$_2$	S3	30-Sep-2019	white cabbage	3	Conventional grower No. 1	DE, 25792, Neuenkirchen	autumn cabbage	marsh soil	Urea, calcium ammonium nitrate, phosphate and potash	QS-Siegel	small, round	outside fresh, brown stem	1.74	September	no	1-2 days 6-8°C, then center of distribution	3 days	7-10 °C day/night in cooling counter
ORG$_1$	S3	1-Oct-2019	red cabbage	1	Organic grower No. 2	DE, 25761, Hedwigenkoog	autumn cabbage, seedfast	sea marsh soil	compost	Demeter, EU organic label	small, round	tight leaves, fresh	1.02	September	no	1-2 days 7-8 °C , then 1-3 days center of distribution, then within 48 h to food retailer	1-3 days	10 °C day/night in cooling counter
ORG$_1$	S3	1-Oct-2019	red cabbage	2	Organic grower No. 2	DE, 25761, Hedwigenkoog	autumn cabbage, seedfast	sea marsh soil	compost	Demeter, EU organic label	small, round	older, white site, partly limp	0.74	September	no	1-2 days 7-8 °C , then 1-3 days center of distribution, then within 48 h to food retailer	1-3 days	10 °C day/night in cooling counter
ORG$_1$	S3	1-Oct-2019	red cabbage	3	Organic grower No. 2	DE, 25761, Hedwigenkoog	autumn cabbage, seedfast	sea marsh soil	compost	Demeter, EU organic label	small, round	fresh with feeding sites	1.03	September	no	1-2 days 7-8 °C , then 1-3 days center of distribution, then within 48 h to food retailer	1-3 days	10 °C day/night in cooling counter
ORG$_1$	S3	1-Oct-2019	white cabbage	1	Organic grower No. 2	DE, 25761, Hedwigenkoog	autumn cabbage, seedfast	sea marsh soil	compost	Demeter, EU organic label	medium-size, round	with holes, slightly damages, loose leaves	1.17	September	no	1-2 days 7-8 °C , then 1-3 days center of distribution, then within 48 h to food retailer	0 days	10 °C day/night in cooling counter
ORG$_1$	S3	1-Oct-2019	white cabbage	2	Organic grower No. 2	DE, 25761, Hedwigenkoog	autumn cabbage, seedfast	sea marsh soil	compost	Demeter, EU organic label	small to medium-size, round	tight leaves, fresh	1.15	September	no	1-2 days 7-8 °C , then 1-3 days center of distribution, then within 48 h to food retailer	0 days	10 °C day/night in cooling counter
ORG$_1$	S3	1-Oct-2019	white cabbage	3	Organic grower No. 2	DE, 25761, Hedwigenkoog	autumn cabbage, seedfast	sea marsh soil	compost	Demeter, EU organic label	very small	some feeding sites	0.81	September	no	1-2 days 7-8 °C , then 1-3 days center of distribution, then within 48 h to food retailer	0 days	10 °C day/night in cooling counter
	IGZ	10-Oct-2019	red cabbage	1	IGZ	DE, 14979, Großbeeren	late cabbage, Redma RZ F1	silty loam	Calcium ammonium nitrate, patentkali	n.a.	medium-size, oval	tight leaves, fresh	1.64	10-Oct-2019	no	not applicable	0 days	not applicable
	IGZ	10-Oct-2019	red cabbage	2	IGZ	DE, 14979, Großbeeren	late cabbage, Redma RZ F2	silty loam	Calcium ammonium nitrate, patentkali	n.a.	medium-size, oval	tight leaves, fresh	1.52	10-Oct-2019	no	not applicable	0 days	not applicable
	IGZ	10-Oct-2019	red cabbage	3	IGZ	DE, 14979, Großbeeren	late cabbage, Redma RZ F3	silty loam	Calcium ammonium nitrate, patentkali	n.a.	medium-size, oval	tight leaves, fresh	1.52	10-Oct-2019	no	not applicable	0 days	not applicable
	IGZ	10-Oct-2019	red cabbage	4	IGZ	DE, 14979, Großbeeren	late cabbage, Redma RZ F4	silty loam	Calcium ammonium nitrate, patentkali	n.a.	medium-size, oval	tight leaves, fresh	1.72	10-Oct-2019	no	not applicable	0 days	not applicable
CON$_1$	S4	21-Oct-2019	red cabbage	1	Conventional grower No. 1	DE, 25792, Neuenkirchen	autumn cabbage	marsh soil	Urea, calcium ammonium nitrate, phosphate and potash	QS-Siegel	big, round	fresh	2.06	October	no	1-2 days 6-8°C, 1-2 days 6°C, then center of distribution	2 days	day and night in vegetable shelf (20 °C with air conditioning)
CON$_1$	S4	21-Oct-2019	red cabbage	2	Conventional grower No. 1	DE, 25792, Neuenkirchen	autumn cabbage	marsh soil	Urea, calcium ammonium nitrate, phosphate and potash	QS-Siegel	medium-size, round	fresh	1.78	October	no	1-2 days 6-8°C, 1-2 days 6°C, then center of distribution	2 days	day and night in vegetable shelf (20 °C with air conditioning)
CON$_1$	S4	21-Oct-2019	red cabbage	3	Conventional grower No. 1	DE, 25792, Neuenkirchen	autumn cabbage	marsh soil	Urea, calcium ammonium nitrate, phosphate and potash	QS-Siegel	small, round	minor feeding sites, fresh	1.61	October	no	1-2 days 6-8°C, 1-2 days 6°C, then center of distribution	2 days	day and night in vegetable shelf (20 °C with air conditioning)
CON$_1$	S4	21-Oct-2019	white cabbage	1	Conventional grower No. 2	DE, 25709, Helse	autumn cabbage, hybrid cultivar	marsh soil	PKS fertilizer (blends), ammonium nitrate and urea (UAN), calcium cyanamide	QS-Siegel	very small, round	fresh	0.87	October	no	1-2 days 6-7°C	2 days	day and night in vegetable shelf (20 °C with air conditioning)
CON$_1$	S4	21-Oct-2019	white cabbage	2	Conventional grower No. 2	DE, 25709, Helse	autumn cabbage, hybrid cultivar	marsh soil	PKS fertilizer (blends), ammonium nitrate and urea (UAN), calcium cyanamide	QS-Siegel	big, round	fresh, with a cut, dirty	2.07	October	no	1-2 days 6-7°C	2 days	day and night in vegetable shelf (20 °C with air conditioning)
CON$_1$	S4	21-Oct-2019	white cabbage	3	Conventional grower No. 2	DE, 25709, Helse	autumn cabbage, hybrid cultivar	marsh soil	PKS fertilizer (blends), ammonium nitrate and urea (UAN), calcium cyanamide	QS-Siegel	medium-size, round	fresh	1.72	October	no	1-2 days 6-7°C	2 days	day and night in vegetable shelf (20 °C with air conditioning)
CON$_2$	S4	21-Oct-2019	red cabbage	1	Conventional grower No. 1	DE, 25792, Neuenkirchen	autumn cabbage	marsh soil	Urea, calcium ammonium nitrate, phosphate and potash	QS-Siegel	medium-size, oval	limp, wrinkly	1.99	October	no	1-2 days 6-8°C, then center of distribution	2 days	7-10 °C day/night in cooling counter
CON$_2$	S4	23-Oct-2019	red cabbage	2	Conventional grower No. 1	DE, 25792, Neuenkirchen	autumn cabbage	marsh soil	Urea, calcium ammonium nitrate, phosphate and potash	QS-Siegel	small, round	fresh, tight leaves	0.80	October	no	1-2 days 6-8°C, then center of distribution	2 days	7-10 °C day/night in cooling counter
CON$_2$	S4	23-Oct-2019	red cabbage	3	Conventional grower No. 1	DE, 25792, Neuenkirchen	autumn cabbage	marsh soil	Urea, calcium ammonium nitrate, phosphate and potash	QS-Siegel	small, round	fresh	1.00	October	no	1-2 days 6-8°C, then center of distribution	2 days	7-10 °C day/night in cooling counter
CON$_2$	S4	21-Oct-2019	white cabbage	1	Conventional grower No. 1	DE, 25792, Neuenkirchen	autumn cabbage	marsh soil	Urea, calcium ammonium nitrate, phosphate and potash	QS-Siegel	medium-size, round	fresh	2.13	October	no	1-2 days 6-8°C, then center of distribution	2 days	7-10 °C day/night in cooling counter
CON$_2$	S4	21-Oct-2019	white cabbage	2	Conventional grower No. 1	DE, 25792, Neuenkirchen	autumn cabbage	marsh soil	Urea, calcium ammonium nitrate, phosphate and potash	QS-Siegel	medium-size, round	slightly limp, damages sites	1.78	October	no	1-2 days 6-8°C, then center of distribution	2 days	7-10 °C day/night in cooling counter
CON$_2$	S4	21-Oct-2019	white cabbage	3	Conventional grower No. 1	DE, 25792, Neuenkirchen	autumn cabbage	marsh soil	Urea, calcium ammonium nitrate, phosphate and potash	QS-Siegel	medium-size, round	fresh	2.26	October	no	1-2 days 6-8°C, then center of distribution	2 days	7-10 °C day/night in cooling counter
ORG$_1$	S4	23-Oct-2019	red cabbage	1	Organic grower No. 2	DE, 25761, Hedwigenkoog	autumn cabbage, seedfast	sea marsh soil	compost	Demeter, EU organic label	small to medium-size, round/bumpy	tight leaves, fresh	0.97	October	no	1-2 days 7-8 °C , then 1-3 days center of distribution, then within 48 h to food retailer	2 days	10 °C day/night in cooling counter
ORG$_1$	S4	23-Oct-2019	red cabbage	2	Organic grower No. 2	DE, 25761, Hedwigenkoog	autumn cabbage, seedfast	sea marsh soil	compost	Demeter, EU organic label	big, round/bumpy	tight leaves, fresh	1.40	October	no	1-2 days 7-8 °C , then 1-3 days center of distribution, then within 48 h to food retailer	2 days	10 °C day/night in cooling counter
ORG$_1$	S4	23-Oct-2019	red cabbage	3	Organic grower No. 2	DE, 25761, Hedwigenkoog	autumn cabbage, seedfast	sea marsh soil	compost	Demeter, EU organic label	small to medium-size, round/bumpy	partly limp	1.09	October	no	1-2 days 7-8 °C , then 1-3 days center of distribution, then within 48 h to food retailer	2 days	10 °C day/night in cooling counter
ORG$_1$	S4	23-Oct-2019	white cabbage	1	Organic grower No. 1	DE, 17237, Blankensee (Mecklenburg)	autumn cabbage, not seedfast	loamy sandy soil	200kg/ha hair-meal pellets	Bioland, EU organic label	small, round	fresh	0.93	October	no	1-2 days 2-6 °C , then 1-3 days 4-7°C, then within 48 h to food retailer	1 day	10 °C day/night in cooling counter
ORG$_1$	S4	23-Oct-2019	white cabbage	2	Organic grower No. 1	DE, 17237, Blankensee (Mecklenburg)	autumn cabbage, not seedfast	loamy sandy soil	200kg/ha hair-meal pellets	Bioland, EU organic label	small, round	fresh	0.84	October	no	1-2 days 2-6 °C , then 1-3 days 4-7°C, then within 48 h to food retailer	1 day	10 °C day/night in cooling counter
ORG$_1$	S4	23-Oct-2019	white cabbage	3	Organic grower No. 1	DE, 17237, Blankensee (Mecklenburg)	autumn cabbage, not seedfast	loamy sandy soil	200kg/ha hair-meal pellets	Bioland, EU organic label	small, round	fresh	0.87	October	no	1-2 days 2-6 °C , then 1-3 days 4-7°C, then within 48 h to food retailer	1 day	10 °C day/night in cooling counter
	IGZ	29-Oct-2019	white cabbage	1	IGZ	DE, 14979, Großbeeren	late cabbage, Dottenfelder Dauer (organic seeds)	silty loam	60 kg N/ha, Vinasse	n.a.	medium-size, round	fresh, slightly dirty	1.69	29-Oct-2019	no	not applicable	0 days	not applicable
	IGZ	29-Oct-2019	white cabbage	2	IGZ	DE, 14979, Großbeeren	late cabbage, Dottenfelder Dauer (organic seeds)	silty loam	60 kg N/ha, Vinasse	n.a.	medium-size, round	fresh	1.13	29-Oct-2019	no	not applicable	0 days	not applicable
	IGZ	29-Oct-2019	white cabbage	3	IGZ	DE, 14979, Großbeeren	late cabbage, Dottenfelder Dauer (organic seeds)	silty loam	60 kg N/ha, Vinasse	n.a.	small, round	fresh, with feeding sites, slightly red	0.90	29-Oct-2019	no	not applicable	0 days	not applicable
	IGZ	29-Oct-2019	white cabbage	4	IGZ	DE, 14979, Großbeeren	late cabbage, Dottenfelder Dauer (organic seeds)	silty loam	60 kg N/ha, Vinasse	n.a.	big, round	fresh, dirty, slightly red	1.95	29-Oct-2019	no	not applicable	0 days	not applicable
CON$_1$	S5	4-Nov-2019	red cabbage	1	Conventional grower No. 2	DE, 25709, Helse	autumn cabbage, hybrid cultivar	marsh soil	PKS fertilizer (blends), ammonium nitrate and urea (UAN), calcium cyanamide	QS-Siegel	big, round	unfresh, dark violett, with a cut	2.43	November	no	1-2 days 6-7°C	2 days	day and night in vegetable shelf (20 °C with air conditioning)
CON$_1$	S5	4-Nov-2019	red cabbage	2	Conventional grower No. 2	DE, 25709, Helse	autumn cabbage, hybrid cultivar	marsh soil	PKS fertilizer (blends), ammonium nitrate and urea (UAN), calcium cyanamide	QS-Siegel	big, oval	unfresh, with a minor feeding site	2.15	November	no	1-2 days 6-7°C	2 days	day and night in vegetable shelf (20 °C with air conditioning)
CON$_1$	S5	4-Nov-2019	red cabbage	3	Conventional grower No. 2	DE, 25709, Helse	autumn cabbage, hybrid cultivar	marsh soil	PKS fertilizer (blends), ammonium nitrate and urea (UAN), calcium cyanamide	QS-Siegel	small, round	cut and feeding site, very old and soft core	1.27	November	no	1-2 days 6-7°C	2 days	day and night in vegetable shelf (20 °C with air conditioning)
CON$_1$	S5	4-Nov-2019	white cabbage	1	Conventional grower No. 1	DE, 25792, Neuenkirchen	late cabbage	marsh soil	Urea, calcium ammonium nitrate, phosphate and potash	QS-Siegel	big, round	fesh, with a feeding site	2.49	November	no	1-2 days 6-8°C, then center of distribution	2 days	day and night in vegetable shelf (20 °C with air conditioning)
CON$_1$	S5	4-Nov-2019	white cabbage	2	Conventional grower No. 1	DE, 25792, Neuenkirchen	late cabbage	marsh soil	Urea, calcium ammonium nitrate, phosphate and potash	QS-Siegel	medium-size, round	slightly damaged, bit violet	2.10	November	no	1-2 days 6-8°C, then center of distribution	2 days	day and night in vegetable shelf (20 °C with air conditioning)
CON$_1$	S5	4-Nov-2019	white cabbage	3	Conventional grower No. 1	DE, 25792, Neuenkirchen	late cabbage	marsh soil	Urea, calcium ammonium nitrate, phosphate and potash	QS-Siegel	medium-size, round	fesh, with a feeding site	1.99	November	no	1-2 days 6-8°C, then center of distribution	2 days	day and night in vegetable shelf (20 °C with air conditioning)
CON$_2$	S5	4-Nov-2019	red cabbage	1	Conventional grower No. 1	DE, 25792, Neuenkirchen	autumn cabbage	marsh soil	Urea, calcium ammonium nitrate, phosphate and potash	QS-Siegel	medium-size, round	fesh, minor feeding sites	1.92	November	no	1-2 days 6-8°C, then center of distribution	2 days	7-10 °C day/night in cooling counter
CON$_2$	S5	4-Nov-2019	red cabbage	2	Conventional grower No. 1	DE, 25792, Neuenkirchen	autumn cabbage	marsh soil	Urea, calcium ammonium nitrate, phosphate and potash	QS-Siegel	medium-size, round	fresh, slightly limp	2.06	November	no	1-2 days 6-8°C, then center of distribution	2 days	7-10 °C day/night in cooling counter
CON$_2$	S5	6-Nov-2019	red cabbage	3	Conventional grower No. 1	DE, 25792, Neuenkirchen	autumn cabbage	marsh soil	Urea, calcium ammonium nitrate, phosphate and potash	QS-Siegel	small, round	very fresh, light violet	0.99	November	no	1-2 days 6-8°C, then center of distribution	1 day	7-10 °C day/night in cooling counter
CON$_2$	S5	4-Nov-2019	white cabbage	1	Conventional grower No. 1	DE, 25792, Neuenkirchen	autumn cabbage	marsh soil	Urea, calcium ammonium nitrate, phosphate and potash	QS-Siegel	medium-size, round	fresh	1.76	November	no	1-2 days 6-8°C, then center of distribution	2 days	7-10 °C day/night in cooling counter
CON$_2$	S5	4-Nov-2019	white cabbage	2	Conventional grower No. 1	DE, 25792, Neuenkirchen	autumn cabbage	marsh soil	Urea, calcium ammonium nitrate, phosphate and potash	QS-Siegel	medium-size, round	fresh, damaged stem, very deep cut	1.74	November	no	1-2 days 6-8°C, then center of distribution	2 days	7-10 °C day/night in cooling counter
CON$_2$	S5	4-Nov-2019	white cabbage	3	Conventional grower No. 1	DE, 25792, Neuenkirchen	autumn cabbage	marsh soil	Urea, calcium ammonium nitrate, phosphate and potash	QS-Siegel	n.a.	unfresh, old, damaged sites and feeding sites, deep cut	1.63	November	no	1-2 days 6-8°C, then center of distribution	2 days	7-10 °C day/night in cooling counter
ORG$_1$	S5	6-Nov-2019	red cabbage	1	Organic grower No. 2	DE, 25761, Hedwigenkoog	autumn cabbage, seedfast	sea marsh soil	compost	Demeter, EU organic label	small, round	frash, dark violett	0.75	November	no	1-2 days 7-8 °C , then 1-3 days center of distribution, then within 48 h to food retailer	2 days	10 °C day/night in cooling counter
ORG$_1$	S5	6-Nov-2019	red cabbage	2	Organic grower No. 2	DE, 25761, Hedwigenkoog	autumn cabbage, seedfast	sea marsh soil	compost	Demeter, EU organic label	medium-size, round	unfresh, light violett, limp, big holes	1.18	November	no	1-2 days 7-8 °C , then 1-3 days center of distribution, then within 48 h to food retailer	2 days	10 °C day/night in cooling counter
ORG$_1$	S5	6-Nov-2019	red cabbage	3	Organic grower No. 2	DE, 25761, Hedwigenkoog	autumn cabbage, seedfast	sea marsh soil	compost	Demeter, EU organic label	medium-size, round	unfresh, light violett, limp	1.23	November	no	1-2 days 7-8 °C , then 1-3 days center of distribution, then within 48 h to food retailer	2 days	10 °C day/night in cooling counter
ORG$_1$	S5	6-Nov-2019	white cabbage	1	Organic grower No. 1	DE, 17237, Blankensee (Mecklenburg)	autumn cabbage, not seedfast	loamy sandy soil	200kg/ha hair-meal pellets	Bioland, EU organic label	very small, round	unfresh	0.79	November	no	1-2 days 2-6 °C , then 1-3 days 4-7°C, then within 48 h to food retailer	2 days	10 °C day/night in cooling counter
ORG$_1$	S5	6-Nov-2019	white cabbage	2	Organic grower No. 1	DE, 17237, Blankensee (Mecklenburg)	autumn cabbage, not seedfast	loamy sandy soil	200kg/ha hair-meal pellets	Bioland, EU organic label	medium-size, round	older leaves, overall fresh	1.33	November	no	1-2 days 2-6 °C , then 1-3 days 4-7°C, then within 48 h to food retailer	2 days	10 °C day/night in cooling counter
ORG$_1$	S5	6-Nov-2019	white cabbage	3	Organic grower No. 1	DE, 17237, Blankensee (Mecklenburg)	autumn cabbage, not seedfast	loamy sandy soil	200kg/ha hair-meal pellets	Bioland, EU organic label	very big, round	unfresh, big hole	1.85	November	no	1-2 days 2-6 °C , then 1-3 days 4-7°C, then within 48 h to food retailer	2 days	10 °C day/night in cooling counter
CON$_1$	S6	18-Nov-2019	red cabbage	1	Conventional grower No. 2	DE, 25709, Helse	autumn cabbage, hybrid cultivar	marsh soil	PKS fertilizer (blends), ammonium nitrate and urea (UAN), calcium cyanamide	QS-Siegel	small, oval	tight dull leaves, fresh	1.18	unknown	yes	refrigerated warehouse 0.1-0.3 °C	2 days	day and night in vegetable shelf (20 °C with air conditioning)
CON$_1$	S6	18-Nov-2019	red cabbage	2	Conventional grower No. 2	DE, 25709, Helse	autumn cabbage, hybrid cultivar	marsh soil	PKS fertilizer (blends), ammonium nitrate and urea (UAN), calcium cyanamide	QS-Siegel	medium-size, round	tight dull leaves, fresh	1.52	unknown	yes	refrigerated warehouse 0.1-0.3 °C	2 days	day and night in vegetable shelf (20 °C with air conditioning)
CON$_1$	S6	18-Nov-2019	red cabbage	3	Conventional grower No. 2	DE, 25709, Helse	autumn cabbage, hybrid cultivar	marsh soil	PKS fertilizer (blends), ammonium nitrate and urea (UAN), calcium cyanamide	QS-Siegel	small, round	tight dull leaves, fresh	1.23	unknown	yes	refrigerated warehouse 0.1-0.3 °C	2 days	day and night in vegetable shelf (20 °C with air conditioning)
CON$_1$	S6	18-Nov-2019	white cabbage	1	Conventional grower No. 1	DE, 25792, Neuenkirchen	late cabbage	marsh soil	Urea, calcium ammonium nitrate, phosphate and potash	QS-Siegel	medium-size, round	fresh, deep cut	1.37	unknown	yes	refrigerated warehouse 0.1-0.3 °C	2 days	day and night in vegetable shelf (20 °C with air conditioning)
CON$_1$	S6	18-Nov-2019	white cabbage	2	Conventional grower No. 1	DE, 25792, Neuenkirchen	late cabbage	marsh soil	Urea, calcium ammonium nitrate, phosphate and potash	QS-Siegel	medium-size, round	tight leaves, fresh	1.62	unknown	yes	refrigerated warehouse 0.1-0.3 °C	2 days	day and night in vegetable shelf (20 °C with air conditioning)
CON$_1$	S6	18-Nov-2019	white cabbage	3	Conventional grower No. 1	DE, 25792, Neuenkirchen	late cabbage	marsh soil	Urea, calcium ammonium nitrate, phosphate and potash	QS-Siegel	medium-size, round	tight leaves, fresh	1.42	unknown	yes	refrigerated warehouse 0.1-0.3 °C	2 days	day and night in vegetable shelf (20 °C with air conditioning)
CON$_2$	S6	18-Nov-2019	red cabbage	1	Conventional grower No. 1	DE, 25792, Neuenkirchen	autumn cabbage	marsh soil	Urea, calcium ammonium nitrate, phosphate and potash	QS-Siegel	medium-size, oval	bit limp, mainly fresh, glossy	1.77	unknown	yes	refrigerated warehouse 0.1-0.3 °C	2 days	7-10 °C day/night in cooling counter
CON$_2$	S6	18-Nov-2019	red cabbage	2	Conventional grower No. 1	DE, 25792, Neuenkirchen	autumn cabbage	marsh soil	Urea, calcium ammonium nitrate, phosphate and potash	QS-Siegel	big, oval	bit limp, mainly fresh, glossy	1.99	unknown	yes	refrigerated warehouse 0.1-0.3 °C	2 days	7-10 °C day/night in cooling counter
CON$_2$	S6	18-Nov-2019	red cabbage	3	Conventional grower No. 1	DE, 25792, Neuenkirchen	autumn cabbage	marsh soil	Urea, calcium ammonium nitrate, phosphate and potash	QS-Siegel	big, round	tight leaves, fresh, glossy	2.59	unknown	yes	refrigerated warehouse 0.1-0.3 °C	2 days	7-10 °C day/night in cooling counter
CON$_2$	S6	18-Nov-2019	white cabbage	1	Conventional grower No. 1	DE, 25792, Neuenkirchen	autumn cabbage	marsh soil	Urea, calcium ammonium nitrate, phosphate and potash	QS-Siegel	medium-size, round	tight leaves, fresh, glossy	1.89	unknown	yes	refrigerated warehouse 0.1-0.3 °C	2 days	7-10 °C day/night in cooling counter
CON$_2$	S6	18-Nov-2019	white cabbage	2	Conventional grower No. 1	DE, 25792, Neuenkirchen	autumn cabbage	marsh soil	Urea, calcium ammonium nitrate, phosphate and potash	QS-Siegel	medium-size, round	tight leaves, fresh	2.10	unknown	yes	refrigerated warehouse 0.1-0.3 °C	2 days	7-10 °C day/night in cooling counter
CON$_2$	S6	18-Nov-2019	white cabbage	3	Conventional grower No. 1	DE, 25792, Neuenkirchen	autumn cabbage	marsh soil	Urea, calcium ammonium nitrate, phosphate and potash	QS-Siegel	medium-size, round	tight leaves, fresh	2.20	unknown	yes	refrigerated warehouse 0.1-0.3 °C	2 days	7-10 °C day/night in cooling counter
ORG$_1$	S6	18-Nov-2019	red cabbage	1	Organic grower No. 3	14715, Seeblick	autumn cabbage, non-hybrid cultivar	loamy sandy soil	compost	Demeter, EU organic label	small, round	fresh	1.02	unknown	yes	refrigerated warehouse 0.1-0.3 °C	4-5 days	10 °C day/night in cooling counter
ORG$_1$	S6	20-Nov-2019	red cabbage	2	Organic grower No. 3	14715, Seeblick	autumn cabbage, non-hybrid cultivar	loamy sandy soil	compost	Demeter, EU organic label	small, round	fresh	1.06	unknown	yes	refrigerated warehouse 0.1-0.3 °C	4-5 days	10 °C day/night in cooling counter
ORG$_1$	S6	20-Nov-2019	red cabbage	3	Organic grower No. 3	14715, Seeblick	autumn cabbage, non-hybrid cultivar	loamy sandy soil	compost	Demeter, EU organic label	small, round	fresh	0.83	unknown	yes	refrigerated warehouse 0.1-0.3 °C	4-5 days	10 °C day/night in cooling counter
ORG$_1$	S6	20-Nov-2019	white cabbage	1	Organic grower No. 4	DE, 15306 Vierlinden	autumn cabbage, hybrid cultivar	loamy sandy soil	hair-meal pellets	Bioland, EU organic label	very big, round	fresh, deep hole	2.39	unknown	yes	refrigerated warehouse 0.1-0.3 °C	4-5 days	10 °C day/night in cooling counter
ORG$_1$	S6	20-Nov-2019	white cabbage	2	Organic grower No. 4	DE, 15306 Vierlinden	autumn cabbage, hybrid cultivar	loamy sandy soil	hair-meal pellets	Bioland, EU organic label	big, round	fresh	1.93	unknown	yes	refrigerated warehouse 0.1-0.3 °C	4-5 days	10 °C day/night in cooling counter
ORG$_1$	S6	20-Nov-2019	white cabbage	3	Organic grower No. 4	DE, 15306 Vierlinden	autumn cabbage, hybrid cultivar	loamy sandy soil	hair-meal pellets	Bioland, EU organic label	big, round	some small holes	1.97	unknown	yes	refrigerated warehouse 0.1-0.3 °C	4-5 days	10 °C day/night in cooling counter
