# Supplementary material for: Seasonal Variation of Glucosinolate Hydrolysis Products in Commercial White and Red Cabbages (Brassica oleracea var. capitata)
Source: Foods. 2020 Nov 17;9(11):1682. doi: 10.3390/foods9111682 (PMC7698573; doi:10.3390/foods9111682)

Figure S1: Allyl glucosinolate hydrolysis product ratios [percentage of allyl isothiocyanate (Allyl-ITC), 3-butenenitrile (Allyl-CN) and 1-cyano-2,3-epithiopropane (CETP)] from white (A-C) and red cabbages (D-F) from conventional (A, B, D, E) and organic supermarkets (C, F) on different sampling dates (S1-S6). Exact sampling dates can be found in Table 1. A) and D) represent conventional supermarket 1 (CON_1), B) and E) stand for conventional supermarket 2 (CON_2) and C) and F) show results from organic supermarket 1 (ORG_1). Each color in the bar of the given bar chart represents the mean minus standard deviation (SD) of the GLS hydrolysis products from three cabbage heads from the same supermarket (n=3). Different lower case letters indicate significant differences in means between the ratios of total ITCs, CNs and ETNs in different samples, as tested by ANOVA and Tukey HSD test at the p ≤ 0.05 level. FW: fresh weight. *Samples were analyzed from 1 g of sample (instead of 0.5 g).

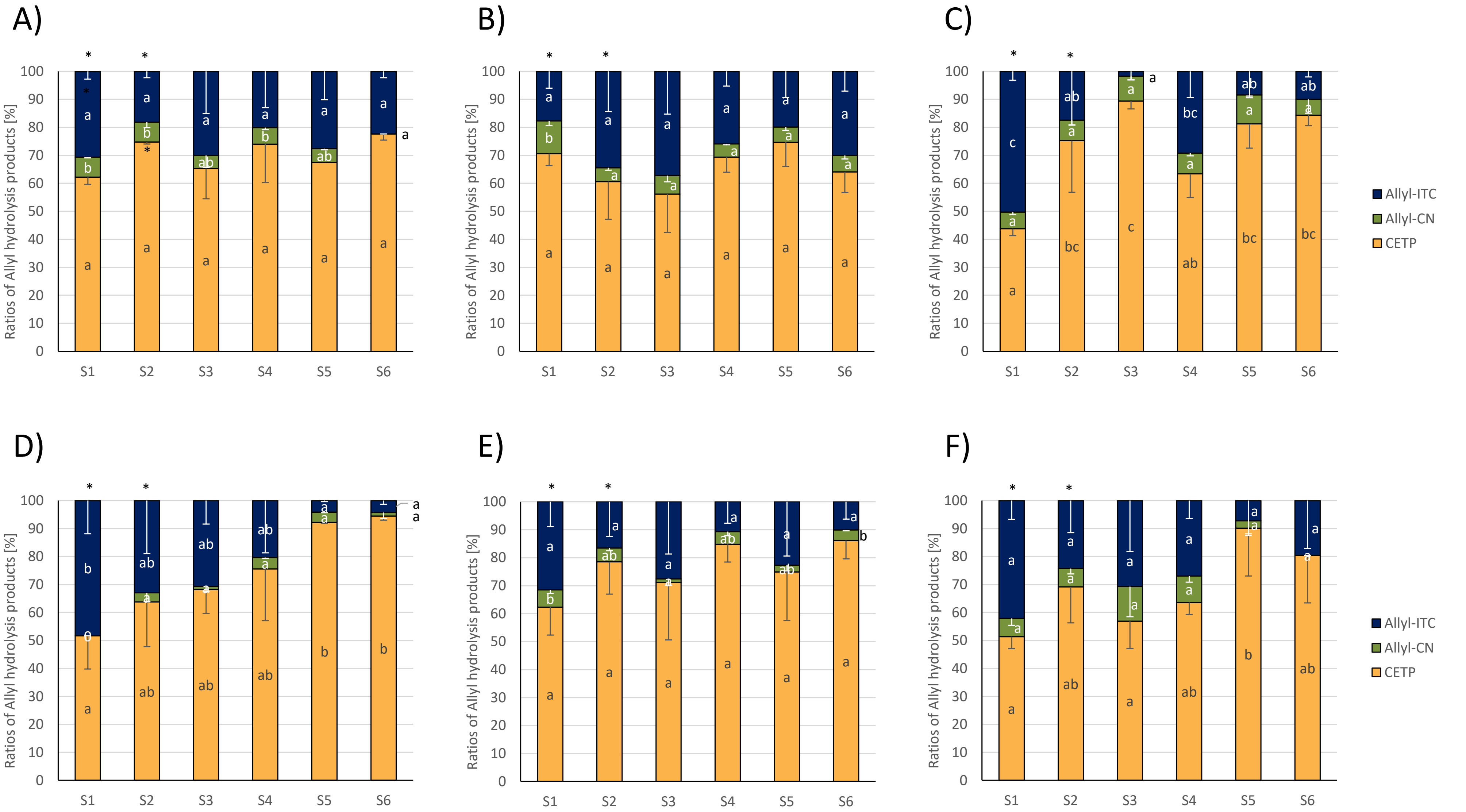

Supplement: Supplementary file 1 [file foods-09-01682-s001.zip › Figure S1.pdf]
