# Supplementary material for: Seasonal Variation of Glucosinolate Hydrolysis Products in Commercial White and Red Cabbages (Brassica oleracea var. capitata)
Source: Foods. 2020 Nov 17;9(11):1682. doi: 10.3390/foods9111682 (PMC7698573; doi:10.3390/foods9111682)
Supplement: Supplementary file 1 [file foods-09-01682-s001.zip › Table S2.pdf]

Table S2: Glucosinolates (GLSs) content in commercial and cabbages harvested freshly from the field in µmol/g fresh weight.

Abbreviations: AV: average; SD: standard deviation; Allyl: allyl GLS; 3But: 3-butenyl GLS; 2OH3But: 2-(R)-2-hydroxy-3-butenyl GLS; 3MTP: 3-(methylsulfanyl)propyl GLS; 3MSOP: 3-(methylsulfinyl)propyl GLS; 4MTB: 4-(methylsulfanyl)butyl GLS; 4MSOB: 4-(methylsulfinyl)butyl GLS; 2PE: 2-phenylethyl GLS; 4OHI3M: 4-hydroxyindol-3-ylmethyl GLS; I3M: indol-3-ylmethyl GLS; 4MOI3M: 4-methoxyindol-3-yl-methyl GLS; 1MOI3M: 1-methoxyindol-3-ylmethyl GLS

From	Variety	Sampling		Allyl	3But	2OH3But	3MTP	3MSOP	4MTB	4MSOB	2PE	4OHI3M	I3M	4MOI3M	1MOI3M
**CON_1**	**white cabbage**	**S1**	**AV**	**0.3331**	**0.0930**	**0.1399**	**0.0286**	**0.1937**	**0.0053**	**0.0993**	**0.0079**	**0.0113**	**0.3119**	**0.0260**	**0.0209**
			**SD**	0.0398	0.0178	0.0203	0.0027	0.0583	0.0017	0.0332	0.0030	0.0042	0.0413	0.0054	0.0064
**CON_1**	**white cabbage**	**S2**	**AV**	**0.5326**	**0.1849**	**0.2121**	**0.0290**	**0.1829**	**0.0046**	**0.0775**	**0.0077**	**0.0160**	**0.2512**	**0.0309**	**0.0309**
			**SD**	0.1835	0.0317	0.0092	0.0092	0.0408	0.0017	0.0285	0.0033	0.0085	0.0294	0.0040	0.0098
**CON_1**	**white cabbage**	**S3**	**AV**	**0.4945**	**0.0488**	**0.1253**	**0.0806**	**0.2479**	**0.0361**	**0.0590**	**0.0266**	**0.0018**	**0.2644**	**0.0410**	**0.0158**
			**SD**	0.1410	0.0252	0.0693	0.0173	0.0290	0.0203	0.0166	0.0032	0.0002	0.0514	0.0083	0.0057
**CON_1**	**white cabbage**	**S4**	**AV**	**0.4838**	**0.0356**	**0.0277**	**0.0368**	**0.4490**	**0.0014**	**0.0138**	**0.0042**	**0.0055**	**0.2410**	**0.0167**	**0.0101**
			**SD**	0.0851	0.0434	0.0092	0.0176	0.0425	0.0007	0.0043	0.0032	0.0038	0.0544	0.0057	0.0069
**CON_1**	**white cabbage**	**S5**	**AV**	**0.7121**	**0.0086**	**0.0255**	**0.0670**	**0.6110**	**0.0020**	**0.0189**	**0.0058**	**0.0188**	**0.2366**	**0.0320**	**0.0122**
			**SD**	0.0200	0.0008	0.0030	0.0135	0.0825	0.0000	0.0035	0.0007	0.0035	0.0436	0.0021	0.0027
**CON_1**	**white cabbage**	**S6**	**AV**	**0.2531**	**0.0548**	**0.1617**	**0.0274**	**0.3696**	**0.0085**	**0.2013**	**0.0046**	**0.0111**	**0.3239**	**0.0271**	**0.0160**
			**SD**	0.0567	0.0126	0.0230	0.0040	0.0176	0.0037	0.0141	0.0010	0.0006	0.0464	0.0057	0.0043
**CON_2**	**white cabbage**	**S1**	**AV**	**0.1668**	**0.0253**	**0.0507**	**0.0237**	**0.2709**	**0.0054**	**0.0662**	**0.0052**	**0.0106**	**0.1873**	**0.0233**	**0.0084**
			**SD**	0.0287	0.0055	0.0184	0.0072	0.0560	0.0033	0.0150	0.0027	0.0017	0.0552	0.0062	0.0023
**CON_2**	**white cabbage**	**S2**	**AV**	**0.3621**	**0.0303**	**0.0409**	**0.0460**	**0.4363**	**0.0155**	**0.0699**	**0.0063**	**0.0205**	**0.0683**	**0.0238**	**0.0069**
			**SD**	0.0652	0.0161	0.0183	0.0132	0.0895	0.0048	0.0362	0.0027	0.0055	0.0075	0.0017	0.0009
**CON_2**	**white cabbage**	**S3**	**AV**	**0.3429**	**0.0249**	**0.0708**	**0.0277**	**0.3426**	**0.0035**	**0.0452**	**0.0076**	**0.0154**	**0.2174**	**0.0384**	**0.0088**
			**SD**	0.2886	0.0118	0.0344	0.0155	0.2121	0.0011	0.0252	0.0020	0.0059	0.0384	0.0020	0.0046
**CON_2**	**white cabbage**	**S4**	**AV**	**0.5646**	**0.0061**	**0.0192**	**0.0480**	**0.4633**	**0.0012**	**0.0122**	**0.0051**	**0.0146**	**0.2108**	**0.0299**	**0.0145**
			**SD**	0.0453	0.0010	0.0030	0.0087	0.0119	0.0002	0.0012	0.0008	0.0056	0.0151	0.0061	0.0024
**CON_2**	**white cabbage**	**S5**	**AV**	**0.4096**	**0.0450**	**0.0809**	**0.0426**	**0.2327**	**0.0034**	**0.0287**	**0.0061**	**0.0250**	**0.2048**	**0.0310**	**0.0118**
			**SD**	0.0542	0.0121	0.0444	0.0092	0.0841	0.0004	0.0174	0.0001	0.0085	0.0679	0.0088	0.0046
**CON_2**	**white cabbage**	**S6**	**AV**	**0.6090**	**0.0928**	**0.1656**	**0.0492**	**0.3791**	**0.0072**	**0.0569**	**0.0062**	**0.0238**	**0.1913**	**0.0310**	**0.0096**
			**SD**	0.0687	0.0195	0.0187	0.0052	0.0208	0.0017	0.0096	0.0005	0.0052	0.0352	0.0080	0.0013
**ORG_1**	**white cabbage**	**S1**	**AV**	**0.4343**	**0.0263**	**0.0565**	**0.0716**	**0.6395**	**0.0089**	**0.0740**	**0.0046**	**0.0198**	**0.2104**	**0.0616**	**0.0074**
			**SD**	0.0981	0.0035	0.0144	0.0243	0.1654	0.0049	0.0250	0.0028	0.0062	0.0158	0.0106	0.0023
**ORG_1**	**white cabbage**	**S2**	**AV**	**0.3254**	**0.0193**	**0.0305**	**0.0385**	**0.3387**	**0.0000**	**0.0327**	**0.0013**	**0.0185**	**0.3133**	**0.0197**	**0.0277**
			**SD**	0.1490	0.0123	0.0126	0.0316	0.2770	0.0000	0.0396	0.0022	0.0098	0.0853	0.0057	0.0108
**ORG_1**	**white cabbage**	**S3**	**AV**	**0.2746**	**0.0313**	**0.0401**	**0.0202**	**0.2971**	**0.0026**	**0.0522**	**0.0066**	**0.0077**	**0.3176**	**0.0156**	**0.0411**
			**SD**	0.0686	0.0112	0.0123	0.0063	0.0520	0.0019	0.0068	0.0019	0.0015	0.0323	0.0017	0.0084
**ORG_1**	**white cabbage**	**S4**	**AV**	**0.3001**	**0.0142**	**0.0538**	**0.0150**	**0.3426**	**0.0017**	**0.0371**	**0.0093**	**0.0042**	**0.4923**	**0.0421**	**0.0335**
			**SD**	0.0252	0.0022	0.0070	0.0047	0.0661	0.0011	0.0094	0.0018	0.0015	0.1229	0.0084	0.0105
**ORG_1**	**white cabbage**	**S5**	**AV**	**0.2372**	**0.0295**	**0.0435**	**0.0204**	**0.3091**	**0.0045**	**0.0566**	**0.0061**	**0.0074**	**0.2475**	**0.0145**	**0.0331**
			**SD**	0.1580	0.0239	0.0344	0.0098	0.1213	0.0051	0.0310	0.0025	0.0012	0.0693	0.0052	0.0070
**ORG_1**	**white cabbage**	**S6**	**AV**	**0.1755**	**0.0211**	**0.0280**	**0.0442**	**0.2496**	**0.0125**	**0.0429**	**0.0060**	**0.0101**	**0.2272**	**0.0119**	**0.0285**
			**SD**	0.0097	0.0018	0.0064	0.0132	0.0208	0.0098	0.0062	0.0015	0.0017	0.0336	0.0013	0.0013
**IGZ**	**white cabbage**	**Dottenfelder Dauer, 29.10.2019**	**AV**	**1.0083**	**0.0129**	**0.0331**	**0.1379**	**1.0107**	**0.0027**	**0.0172**	**0.0043**	**0.0183**	**0.1854**	**0.0592**	**0.0242**
			**SD**	0.1846	0.0007	0.0175	0.0734	0.2371	0.0010	0.0071	0.0023	0.0050	0.0619	0.0124	0.0082
**CON_1**	**red cabbage**	**S1**	**AV**	**0.2703**	**0.4966**	**0.9892**	**0.0208**	**0.1906**	**0.0751**	**0.9259**	**0.0149**	**0.0337**	**0.2474**	**0.0392**	**0.0086**
			**SD**	0.0379	0.0903	0.1870	0.0046	0.0253	0.0105	0.0545	0.0026	0.0021	0.0541	0.0078	0.0018
**CON_1**	**red cabbage**	**S2**	**AV**	**0.2147**	**0.2807**	**0.3993**	**0.0242**	**0.2529**	**0.0621**	**0.3803**	**0.0052**	**0.0465**	**0.2162**	**0.0397**	**0.0079**
			**SD**	0.0788	0.3613	0.5348	0.0021	0.0399	0.0761	0.3587	0.0090	0.0091	0.0611	0.0096	0.0024
**CON_1**	**red cabbage**	**S3**	**AV**	**0.2978**	**0.2696**	**0.3531**	**0.0293**	**0.3115**	**0.0623**	**0.3351**	**0.0107**	**0.0391**	**0.2204**	**0.0458**	**0.0045**
			**SD**	0.1406	0.2494	0.3253	0.0172	0.2297	0.0254	0.1936	0.0034	0.0163	0.0479	0.0136	0.0008
**CON_1**	**red cabbage**	**S4**	**AV**	**0.2619**	**0.1266**	**0.1579**	**0.0269**	**0.3974**	**0.0385**	**0.2970**	**0.0069**	**0.0381**	**0.2312**	**0.0419**	**0.0054**
			**SD**	0.0610	0.0244	0.0312	0.0050	0.1478	0.0082	0.1204	0.0011	0.0129	0.0081	0.0105	0.0014
**CON_1**	**red cabbage**	**S5**	**AV**	**0.3283**	**0.3947**	**0.8451**	**0.0152**	**0.2315**	**0.0667**	**0.5154**	**0.0079**	**0.0179**	**0.1451**	**0.0205**	**0.0044**
			**SD**	0.0674	0.0507	0.1102	0.0042	0.0450	0.0368	0.1504	0.0018	0.0043	0.0161	0.0049	0.0030
**CON_1**	**red cabbage**	**S6**	**AV**	**0.2749**	**0.3380**	**0.6089**	**0.0176**	**0.2151**	**0.1122**	**0.6803**	**0.0113**	**0.0198**	**0.3268**	**0.0331**	**0.0164**
			**SD**	0.0356	0.0510	0.0451	0.0023	0.0375	0.0195	0.0663	0.0011	0.0028	0.0381	0.0050	0.0090
**CON_2**	**red cabbage**	**S1**	**AV**	**0.2870**	**0.2079**	**0.2267**	**0.0292**	**0.2792**	**0.0729**	**0.3320**	**0.0110**	**0.0135**	**0.1640**	**0.0272**	**0.0083**
			**SD**	0.0273	0.1304	0.0872	0.0164	0.0421	0.0538	0.0814	0.0026	0.0043	0.1043	0.0048	0.0041
**CON_2**	**red cabbage**	**S2**	**AV**	**0.1673**	**0.0584**	**0.0843**	**0.0151**	**0.1653**	**0.0186**	**0.0969**	**0.0000**	**0.0250**	**0.2407**	**0.0339**	**0.0087**
			**SD**	0.0070	0.0239	0.0035	0.0043	0.0493	0.0078	0.0353	0.0000	0.0010	0.0753	0.0029	0.0016
**CON_2**	**red cabbage**	**S3**	**AV**	**0.1847**	**0.3671**	**0.4020**	**0.0104**	**0.1330**	**0.0309**	**0.4235**	**0.0137**	**0.0237**	**0.2428**	**0.0348**	**0.0098**
			**SD**	0.0932	0.2695	0.2511	0.0056	0.0475	0.0185	0.0884	0.0027	0.0145	0.1718	0.0017	0.0082
**CON_2**	**red cabbage**	**S4**	**AV**	**0.2265**	**0.1443**	**0.1774**	**0.0083**	**0.1888**	**0.0143**	**0.2931**	**0.0056**	**0.0187**	**0.2095**	**0.0291**	**0.0048**
			**SD**	0.0735	0.0599	0.1462	0.0027	0.1135	0.0051	0.2303	0.0018	0.0189	0.0366	0.0235	0.0014
**CON_2**	**red cabbage**	**S5**	**AV**	**0.3504**	**0.3254**	**0.5161**	**0.0156**	**0.1687**	**0.0393**	**0.3213**	**0.0095**	**0.0221**	**0.1372**	**0.0285**	**0.0028**
			**SD**	0.1614	0.2206	0.3374	0.0071	0.0567	0.0256	0.1463	0.0039	0.0086	0.0647	0.0090	0.0007
**CON_2**	**red cabbage**	**S6**	**AV**	**0.2946**	**0.1373**	**0.2144**	**0.0197**	**0.2001**	**0.0469**	**0.2049**	**0.0109**	**0.0218**	**0.1431**	**0.0576**	**0.0050**
			**SD**	0.0173	0.0555	0.0668	0.0023	0.0427	0.0279	0.0703	0.0005	0.0024	0.0106	0.0270	0.0029
**ORG_1**	**red cabbage**	**S1**	**AV**	**0.1928**	**0.0473**	**0.0969**	**0.0165**	**0.1928**	**0.0075**	**0.1649**	**0.0053**	**0.0069**	**0.3483**	**0.0335**	**0.0115**
			**SD**	0.0198	0.0026	0.0236	0.0030	0.0284	0.0025	0.0339	0.0013	0.0012	0.0952	0.0086	0.0004
**ORG_1**	**red cabbage**	**S2**	**AV**	**0.2452**	**0.0642**	**0.1518**	**0.0155**	**0.2064**	**0.0065**	**0.2061**	**0.0028**	**0.0099**	**0.4924**	**0.0387**	**0.0109**
			**SD**	0.0307	0.0306	0.0297	0.0006	0.0381	0.0025	0.0385	0.0048	0.0004	0.0649	0.0019	0.0018
**ORG_1**	**red cabbage**	**S3**	**AV**	**0.2698**	**0.2289**	**0.3942**	**0.0129**	**0.2319**	**0.0176**	**0.3442**	**0.0080**	**0.0284**	**0.3409**	**0.0272**	**0.0115**
			**SD**	0.2182	0.3188	0.5544	0.0083	0.0921	0.0116	0.3070	0.0042	0.0069	0.1070	0.0029	0.0062
**ORG_1**	**red cabbage**	**S4**	**AV**	**0.1663**	**0.0545**	**0.0393**	**0.0151**	**0.1447**	**0.0165**	**0.1007**	**0.0060**	**0.0145**	**0.2290**	**0.0279**	**0.0100**
			**SD**	0.0468	0.0262	0.0266	0.0078	0.0730	0.0193	0.0880	0.0033	0.0065	0.0539	0.0101	0.0047
**ORG_1**	**red cabbage**	**S5**	**AV**	**0.0695**	**0.0688**	**0.0956**	**0.0127**	**0.1226**	**0.0199**	**0.1888**	**0.0028**	**0.0098**	**0.3434**	**0.0140**	**0.0169**
			**SD**	0.0363	0.0229	0.0342	0.0028	0.1107	0.0061	0.0506	0.0018	0.0094	0.0649	0.0054	0.0071
**ORG_1**	**red cabbage**	**S6**	**AV**	**0.0431**	**0.0715**	**0.0799**	**0.0119**	**0.0573**	**0.0206**	**0.1673**	**0.0030**	**0.0059**	**0.2832**	**0.0128**	**0.0079**
			**SD**	0.0121	0.0198	0.0222	0.0034	0.0234	0.0071	0.0558	0.0010	0.0016	0.0492	0.0028	0.0023
**IGZ**	**red cabbage**	**Redma RZ F1, 10.10.2019**	**AV**	**0.2876**	**0.5185**	**0.9397**	**0.0101**	**0.1765**	**0.0285**	**1.1194**	**0.0141**	**0.0290**	**0.3250**	**0.0284**	**0.0095**
			**SD**	0.0322	0.0444	0.0812	0.0009	0.0265	0.0035	0.1551	0.0020	0.0059	0.0614	0.0030	0.0020
