# Supplementary material for: Seasonal Variation of Glucosinolate Hydrolysis Products in Commercial White and Red Cabbages (Brassica oleracea var. capitata)
Source: Foods. 2020 Nov 17;9(11):1682. doi: 10.3390/foods9111682 (PMC7698573; doi:10.3390/foods9111682)
Supplement: Supplementary file 1 [file foods-09-01682-s001.zip › Table S3.pdf]

Table S3: Glucosinolate (GLS) hydrolysis product formation in commercial and cabbages harvested freshly from the field in µmol/g fresh weight.

Abbreviations: AV: average; SD: standard deviation; ITC: isothiocyanate; CN: cyanide; ETN: epithionitrile; Allyl: allyl GLS; 3But: 3-butenyl GLS; 2OH3But: 2-hydroxy-3-butenyl GLS; 3MTP: 3-(methylsulfanyl)propyl GLS; 3MSOP: 3-(methylsulfinyl)propyl GLS; 4MTB: 4-(methylsulfanyl)butyl GLS; 4MSOB: 4-(methylsulfinyl)butyl GLS; 2PE: 2-phenylethyl GLS; I3M: indol-3-ylmethyl GLS; Allyl-CN: 3-butenenitrile; Allyl-ITC: allyl ITC; CEPT: 1-cyano-2,3-epithiopropane; 3But-CN: 4-pentenenitrile; 3But-ITC: 3-butenyl ITC; CETB: 1-cyano-3,4-epithiobutane; 2OH3But-CN: 3-hydroxypentenenitrile; CHETB A/B: 1-cyano-2-hydroxy-3,4-epithiobutane A/B; OZT: 5-vinyloxazolidine-2-thione; 3MTP-CN: 4-(methylsulfanyl)butanenitrile; 3MTP-ITC 3-(methylsulfanyl)propyl ITC; 4MTB-CN: 5-(methylsulfanyl)pentanenitrile; 4MTB-ITC: 4-(methylsulfanyl)butyl ITC; 3MSOP-CN: 4-(methylsulfinyl)butanenitrile; 3MSOP-ITC: 3-(methylsulfinyl)propyl ITC; 4MSOB-CN: 5-(methylsulfinyl)pentanenitrile; 4MSOB-ITC: 4-(methylsulfinyl)butyl ITC; 5MSOP-ITC: 5-(methylsulfinyl)pentyl ITC; 2-PE-CN: 3-phenylpropanenitrile; 2-PE-ITC: 2-phenylethyl ITC; IAN: indole-3-acetonitrile

			precursor GLS	Allyl			3But		
			µmol/g fresh weight	Allyl-CN	Allyl-ITC	CETP	3But-CN	3But-ITC	CETB
CON$_1$	white cabba	S1	AV	0.01404	0.05939	0.12166	0.00206	0.00566	0.01438
			SD	0.00178	0.00189	0.01659	0.00051	0.00063	0.00353
CON$_1$	white cabba	S2	AV	0.01823	0.04375	0.18407	0.00349	0.00400	0.02108
			SD	0.00846	0.00695	0.04796	0.00106	0.00252	0.00389
CON$_1$	white cabba	S3	AV	0.00764	0.04498	0.10286	0.00000	0.00209	0.00200
			SD	0.00664	0.01727	0.03308	0.00000	0.00146	0.00192
CON$_1$	white cabba	S4	AV	0.02076	0.07050	0.25671	0.00000	0.00076	0.00148
			SD	0.00359	0.04782	0.04600	0.00000	0.00066	0.00132
CON$_1$	white cabba	S5	AV	0.02336	0.13084	0.31801	0.00000	0.00122	0.00070
			SD	0.00477	0.05376	0.05526	0.00000	0.00059	0.00121
CON$_1$	white cabba	S6	AV	0.00000	0.02891	0.10006	0.00000	0.00438	0.00830
			SD	0.00000	0.00597	0.01376	0.00000	0.00145	0.00277
CON$_2$	white cabba	S1	AV	0.01952	0.02924	0.11782	0.00177	0.00130	0.00575
			SD	0.00444	0.00924	0.02261	0.00040	0.00043	0.00139
CON$_2$	white cabba	S2	AV	0.01136	0.07530	0.14178	0.00000	0.00391	0.00433
			SD	0.00354	0.01771	0.05001	0.00000	0.00122	0.00211
CON$_2$	white cabba	S3	AV	0.01049	0.08264	0.09461	0.00000	0.00274	0.00154
			SD	0.00359	0.08894	0.04310	0.00000	0.00091	0.00266
CON$_2$	white cabba	S4	AV	0.01745	0.09594	0.25613	0.00000	0.00109	0.00090
			SD	0.00180	0.02204	0.01510	0.00000	0.00045	0.00157
CON$_2$	white cabba	S5	AV	0.01459	0.05226	0.19858	0.00000	0.00366	0.00700
			SD	0.00305	0.02409	0.03017	0.00000	0.00323	0.00183
CON$_2$	white cabba	S6	AV	0.02017	0.10301	0.22649	0.00000	0.01246	0.01298
			SD	0.00352	0.01528	0.05641	0.00000	0.00280	0.00590
ORG$_1$	white cabba	S1	AV	0.02010	0.16955	0.14869	0.00000	0.00282	0.00326
			SD	0.00433	0.02444	0.03080	0.00000	0.00057	0.00049
ORG$_1$	white cabba	S2	AV	0.01787	0.04299	0.17665	0.00000	0.00202	0.00339
			SD	0.00795	0.04322	0.05028	0.00000	0.00224	0.00228
ORG$_1$	white cabba	S3	AV	0.00844	0.00171	0.08527	0.00000	0.00000	0.00177
			SD	0.00095	0.00297	0.00435	0.00000	0.00000	0.00307
ORG$_1$	white cabba	S4	AV	0.01593	0.05540	0.13777	0.00000	0.00155	0.00241
			SD	0.00925	0.00707	0.08168	0.00000	0.00036	0.00417
ORG$_1$	white cabba	S5	AV	0.01193	0.01198	0.09285	0.00000	0.00109	0.00374
			SD	0.00381	0.01342	0.02182	0.00000	0.00189	0.00148
ORG$_1$	white cabba	S6	AV	0.00675	0.00994	0.08699	0.00000	0.00030	0.00466
			SD	0.00587	0.00120	0.02426	0.00000	0.00026	0.00284
IGZ	white cabba	Dottenfelder Dauer, 29.10.2019	AV	0.03823	0.17136	0.52926	0.00079	0.00149	0.00304
			SD	0.01223	0.06022	0.19029	0.00159	0.00059	0.00248
CON$_1$	red cabbage	S1	AV	0.00000	0.05993	0.06142	0.00392	0.05502	0.07809
			SD	0.00000	0.02805	0.01969	0.00169	0.05675	0.04642
CON$_1$	red cabbage	S2	AV	0.00325	0.03410	0.06444	0.00189	0.04860	0.03074
			SD	0.00333	0.02369	0.01918	0.00194	0.07633	0.03207
CON$_1$	red cabbage	S3	AV	0.00200	0.03241	0.07177	0.00301	0.03239	0.06545
			SD	0.00346	0.02088	0.04397	0.00521	0.04884	0.10516
CON$_1$	red cabbage	S4	AV	0.00979	0.04561	0.18400	0.00000	0.01430	0.03173
			SD	0.00177	0.03614	0.06380	0.00000	0.01363	0.01498
CON$_1$	red cabbage	S5	AV	0.01254	0.01355	0.30916	0.01260	0.00676	0.27225
			SD	0.00403	0.00297	0.08793	0.00373	0.00177	0.06262
CON$_1$	red cabbage	S6	AV	0.00334	0.00979	0.22317	0.01136	0.00599	0.20078
			SD	0.00578	0.00227	0.02055	0.00166	0.00197	0.01512
CON$_2$	red cabbage	S1	AV	0.01394	0.07089	0.14247	0.00600	0.01519	0.07804
			SD	0.00194	0.01643	0.03175	0.00379	0.01650	0.06121
CON$_2$	red cabbage	S2	AV	0.00459	0.01361	0.07233	0.00000	0.00296	0.00785
			SD	0.00162	0.00852	0.02314	0.00000	0.00198	0.00420
CON$_2$	red cabbage	S3	AV	0.00226	0.04291	0.09209	0.00000	0.04470	0.09008
			SD	0.00391	0.03518	0.00758	0.00000	0.03410	0.03866
CON$_2$	red cabbage	S4	AV	0.00969	0.02511	0.19087	0.00604	0.00785	0.04610
			SD	0.00372	0.01790	0.08325	0.00524	0.00609	0.03901
CON$_2$	red cabbage	S5	AV	0.01004	0.05770	0.27060	0.00972	0.03772	0.20746
			SD	0.00877	0.01776	0.15591	0.00591	0.01257	0.16777
CON$_2$	red cabbage	S6	AV	0.00988	0.02608	0.22267	0.00393	0.00848	0.04754
			SD	0.00138	0.01672	0.02598	0.00171	0.00743	0.02605
ORG$_1$	red cabbage	S1	AV	0.00741	0.05103	0.06026	0.00000	0.00365	0.00429
			SD	0.00120	0.01816	0.00884	0.00000	0.00157	0.00057
ORG$_1$	red cabbage	S2	AV	0.02027	0.07700	0.19409	0.00000	0.00941	0.01230
			SD	0.01220	0.05316	0.02488	0.00000	0.00640	0.00090
ORG$_1$	red cabbage	S3	AV	0.00306	0.01123	0.01814	0.00000	0.00354	0.00032
			SD	0.00273	0.01086	0.00659	0.00000	0.00254	0.00056
ORG$_1$	red cabbage	S4	AV	0.01304	0.03898	0.08958	0.00000	0.00341	0.00267
			SD	0.00306	0.01575	0.02362	0.00000	0.00233	0.00231
ORG$_1$	red cabbage	S5	AV	0.00424	0.01136	0.06047	0.00582	0.00524	0.02806
			SD	0.00734	0.01968	0.04405	0.00083	0.00415	0.00833
ORG$_1$	red cabbage	S6	AV	0.00000	0.00595	0.02047	0.00339	0.00524	0.01267
			SD	0.00000	0.00515	0.00318	0.00028	0.00329	0.00182
IGZ	red cabbage	Redma RZ F1, 10.10.2019	AV	0.00931	0.01508	0.19939	0.01660	0.02248	0.21950
			SD	0.00083	0.00940	0.01926	0.00219	0.01429	0.04469

			precursor GLS	2OH3But				3MTP		4MTB		3MSOP		4MSOB	
			µmol/g fresh weight	2OH3But-CN	CHETB A	CHETB B	OZT	3MTP-CN	3MTP-ITC	4MTB-CN	4MTB-ITC	3MSOP-CN	3MSOP-ITC	4MSOB-CN	4MSOB-ITC
CON$_1$	white cabba	S1	AV	0.01320	0.02204	0.02594	0.00068	0.00165	0.00148	0.00000	0.00074	0.03217	0.17284	0.02280	0.06201
			SD	0.01143	0.00370	0.00389	0.00050	0.00028	0.00037	0.00000	0.00016	0.00347	0.04012	0.00568	0.01229
CON$_1$	white cabba	S2	AV	0.00982	0.03020	0.03837	0.00560	0.00229	0.00086	0.00040	0.00092	0.04040	0.10612	0.04415	0.05549
			SD	0.01700	0.00670	0.00838	0.00040	0.00078	0.00016	0.00041	0.00049	0.00217	0.00213	0.01966	0.02375
CON$_1$	white cabba	S3	AV	0.00000	0.00495	0.00540	0.00000	0.00359	0.00306	0.00047	0.00283	0.06366	0.09861	0.01896	0.01657
			SD	0.00000	0.00553	0.00481	0.00000	0.00078	0.00329	0.00081	0.00139	0.02868	0.03826	0.01331	0.01104
CON$_1$	white cabba	S4	AV	0.00359	0.00325	0.00375	0.00000	0.00843	0.00438	0.00000	0.00000	0.36887	0.39691	0.00823	0.00000
			SD	0.00621	0.00280	0.00087	0.00000	0.00131	0.00339	0.00000	0.00000	0.03392	0.08656	0.00063	0.00000
CON$_1$	white cabba	S5	AV	0.00000	0.00111	0.00000	0.00000	0.00879	0.00615	0.00000	0.00000	0.30269	0.35617	0.00593	0.00172
			SD	0.00000	0.00192	0.00000	0.00000	0.00194	0.00321	0.00000	0.00000	0.08085	0.13268	0.00276	0.00298
CON$_1$	white cabba	S6	AV	0.00000	0.03322	0.03979	0.01056	0.00203	0.00136	0.00047	0.00119	0.18988	0.06964	0.20880	0.03417
			SD	0.00000	0.00954	0.00795	0.00156	0.00032	0.00008	0.00082	0.00021	0.01586	0.00627	0.04474	0.00259
CON$_2$	white cabba	S1	AV	0.00000	0.01158	0.01016	0.00000	0.00300	0.00128	0.00000	0.00053	0.13059	0.17801	0.05049	0.01927
			SD	0.00000	0.00203	0.00288	0.00000	0.00058	0.00022	0.00000	0.00006	0.01301	0.06701	0.00662	0.02797
CON$_2$	white cabba	S2	AV	0.00000	0.00514	0.00367	0.00261	0.00502	0.00582	0.00108	0.00282	0.11128	0.37390	0.03063	0.05991
			SD	0.00000	0.00219	0.00281	0.00227	0.00215	0.00129	0.00060	0.00132	0.04257	0.05591	0.02038	0.01304
CON$_2$	white cabba	S3	AV	0.00000	0.00363	0.00506	0.00000	0.00245	0.00410	0.00000	0.00064	0.10655	0.25338	0.01689	0.01369
			SD	0.00000	0.00629	0.00877	0.00000	0.00215	0.00430	0.00000	0.00015	0.04777	0.23127	0.01346	0.00957
CON$_2$	white cabba	S4	AV	0.00000	0.00384	0.00498	0.00000	0.00668	0.00538	0.00000	0.00091	0.27194	0.27703	0.00955	0.00000
			SD	0.00000	0.00072	0.00062	0.00000	0.00094	0.00136	0.00000	0.00010	0.01901	0.05621	0.00993	0.00000
CON$_2$	white cabba	S5	AV	0.00446	0.01370	0.01903	0.00000	0.00546	0.00302	0.00000	0.00071	0.10113	0.08360	0.00903	0.00381
			SD	0.00772	0.00398	0.00478	0.00000	0.00287	0.00320	0.00000	0.00005	0.01590	0.03194	0.00325	0.00382
CON$_2$	white cabba	S6	AV	0.00000	0.02821	0.02450	0.02004	0.00508	0.00448	0.00000	0.00098	0.16093	0.16703	0.03258	0.01036
			SD	0.00000	0.00825	0.00627	0.00609	0.00205	0.00080	0.00000	0.00085	0.06550	0.00922	0.02292	0.00905
ORG$_1$	white cabba	S1	AV	0.00000	0.00552	0.00566	0.00414	0.00788	0.02256	0.00043	0.00478	0.11185	0.69954	0.01312	0.05316
			SD	0.00000	0.00150	0.00124	0.00105	0.00255	0.00773	0.00039	0.00235	0.01468	0.09507	0.00335	0.01473
ORG$_1$	white cabba	S2	AV	0.00000	0.00299	0.00238	0.00000	0.00553	0.00261	0.00000	0.00014	0.18783	0.50072	0.03624	0.04241
			SD	0.00000	0.00197	0.00116	0.00000	0.00473	0.00359	0.00000	0.00025	0.23167	0.54334	0.05907	0.06510
ORG$_1$	white cabba	S3	AV	0.00000	0.00121	0.00000	0.00000	0.00232	0.00000	0.00000	0.00000	0.08210	0.02601	0.02013	0.00000
			SD	0.00000	0.00209	0.00000	0.00000	0.00014	0.00000	0.00000	0.00000	0.02207	0.00847	0.00920	0.00000
ORG$_1$	white cabba	S4	AV	0.00000	0.00736	0.00986	0.00000	0.00388	0.00251	0.00000	0.00000	0.23745	0.40815	0.03255	0.02380
			SD	0.00000	0.00292	0.00338	0.00000	0.00260	0.00067	0.00000	0.00000	0.09250	0.11649	0.01860	0.01691
ORG$_1$	white cabba	S5	AV	0.00000	0.00720	0.00968	0.00000	0.00238	0.00047	0.00039	0.00061	0.21019	0.02520	0.05244	0.00124
			SD	0.00000	0.00525	0.00285	0.00000	0.00097	0.00046	0.00068	0.00055	0.02447	0.01638	0.03373	0.00215
ORG$_1$	white cabba	S6	AV	0.00000	0.00184	0.00291	0.00000	0.00834	0.00000	0.00167	0.00000	0.16126	0.00998	0.02524	0.00000
			SD	0.00000	0.00318	0.00505	0.00000	0.00477	0.00000	0.00148	0.00000	0.05037	0.00427	0.01287	0.00000
IGZ	white cabba	Dottenfelder Dauer, 29.10.2019	AV	0.00000	0.00157	0.00120	0.00000	0.02696	0.01287	0.00010	0.00000	0.49942	0.27363	0.00353	0.00000
			SD	0.00000	0.00219	0.00186	0.00000	0.01685	0.00671	0.00027	0.00000	0.21583	0.07921	0.00314	0.00000
CON$_1$	red cabbage	S1	AV	0.05760	0.07559	0.08413	0.27407	0.00198	0.00122	0.00977	0.02878	0.00612	0.20881	0.17020	1.05915
			SD	0.00484	0.01600	0.01621	0.13821	0.00096	0.00211	0.00610	0.02066	0.00091	0.03739	0.01742	0.24959
CON$_1$	red cabbage	S2	AV	0.00000	0.02407	0.02878	0.06971	0.00293	0.00235	0.00548	0.02605	0.04659	0.23589	0.09869	0.45429
			SD	0.00000	0.02502	0.02869	0.12074	0.00189	0.00181	0.00651	0.03940	0.02210	0.02726	0.07055	0.52352
CON$_1$	red cabbage	S3	AV	0.00000	0.04145	0.05227	0.02232	0.00239	0.00119	0.00555	0.00821	0.05845	0.12346	0.14453	0.20216
			SD	0.00000	0.06704	0.08309	0.03203	0.00097	0.00049	0.00566	0.00766	0.01230	0.06881	0.16344	0.21420
CON$_1$	red cabbage	S4	AV	0.00000	0.04106	0.02472	0.01275	0.00591	0.00311	0.00592	0.00622	0.31873	0.21356	0.40817	0.17626
			SD	0.00000	0.00907	0.01188	0.01107	0.00292	0.00246	0.00299	0.00414	0.05426	0.07862	0.10331	0.06313
CON$_1$	red cabbage	S5	AV	0.14391	0.28955	0.32322	0.02693	0.00668	0.00037	0.03214	0.00548	0.17962	0.02703	0.89755	0.11789
			SD	0.02370	0.04768	0.06001	0.00413	0.00303	0.00025	0.02238	0.00357	0.05664	0.00705	0.35433	0.04928
CON$_1$	red cabbage	S6	AV	0.00000	0.17267	0.18976	0.02497	0.00606	0.00000	0.03290	0.00303	0.15392	0.02822	1.11431	0.10511
			SD	0.00000	0.02750	0.02432	0.00675	0.00179	0.00000	0.01587	0.00268	0.03455	0.01348	0.17985	0.04781
CON$_2$	red cabbage	S1	AV	0.00000	0.05130	0.05988	0.01556	0.00623	0.00212	0.01278	0.01446	0.05812	0.23008	0.23575	0.31943
			SD	0.00000	0.02663	0.03052	0.00438	0.00421	0.00086	0.01070	0.00802	0.04766	0.11275	0.13373	0.05542
CON$_2$	red cabbage	S2	AV	0.00278	0.01472	0.01925	0.00515	0.00288	0.00055	0.00202	0.00235	0.05362	0.13649	0.07053	0.10895
			SD	0.00481	0.00078	0.00211	0.00569	0.00159	0.00047	0.00104	0.00139	0.02296	0.06145	0.03478	0.06028
CON$_2$	red cabbage	S3	AV	0.00000	0.07784	0.09315	0.03809	0.00151	0.00094	0.00447	0.00494	0.04238	0.09359	0.25098	0.33435
			SD	0.00000	0.03266	0.03763	0.02129	0.00137	0.00073	0.00310	0.00337	0.00342	0.08749	0.08343	0.17963
CON$_2$	red cabbage	S4	AV	0.00000	0.06229	0.06876	0.01589	0.00216	0.00044	0.00158	0.00127	0.19697	0.08520	0.55538	0.10133
			SD	0.00000	0.04096	0.03808	0.01380	0.00055	0.00076	0.00151	0.00119	0.04590	0.06724	0.31222	0.05252
CON$_2$	red cabbage	S5	AV	0.04713	0.17915	0.19973	0.03815	0.00613	0.00195	0.01581	0.00978	0.15114	0.05824	0.55354	0.13624
			SD	0.08163	0.11876	0.12398	0.02187	0.00461	0.00044	0.01429	0.00483	0.05452	0.02478	0.22204	0.09598
CON$_2$	red cabbage	S6	AV	0.00000	0.06166	0.07232	0.01356	0.00562	0.00116	0.00853	0.00401	0.14549	0.03168	0.30172	0.03169
			SD	0.00000	0.02559	0.02942	0.01225	0.00231	0.00107	0.00618	0.00360	0.01931	0.02253	0.22443	0.01856
ORG$_1$	red cabbage	S1	AV	0.00000	0.01630	0.02053	0.00964	0.00166	0.00207	0.00137	0.00806	0.04816	0.28085	0.07522	0.23890
			SD	0.00000	0.00130	0.00062	0.00154	0.00086	0.00184	0.00080	0.00647	0.00186	0.03871	0.00500	0.00588
ORG$_1$	red cabbage	S2	AV	0.00000	0.02617	0.03321	0.00717	0.00252	0.00071	0.00181	0.00361	0.12022	0.46579	0.21410	0.44074
			SD	0.00000	0.00457	0.00908	0.00768	0.00103	0.00082	0.00091	0.00257	0.01557	0.15135	0.02010	0.16274
ORG$_1$	red cabbage	S3	AV	0.00000	0.00000	0.00000	0.00000	0.00000	0.00000	0.00000	0.00000	0.01133	0.03956	0.03994	0.06124
			SD	0.00000	0.00000	0.00000	0.00000	0.00000	0.00000	0.00000	0.00000	0.00244	0.02694	0.04755	0.06484
ORG$_1$	red cabbage	S4	AV	0.00000	0.00859	0.01064	0.00929	0.00283	0.00248	0.00111	0.00329	0.13075	0.13794	0.15618	0.10246
			SD	0.00000	0.00801	0.01103	0.01092	0.00128	0.00159	0.00097	0.00202	0.08876	0.09755	0.18103	0.08934
ORG$_1$	red cabbage	S5	AV	0.00000	0.02025	0.02083	0.00308	0.00234	0.00050	0.00283	0.00173	0.15299	0.10624	0.44825	0.12925
			SD	0.00000	0.00382	0.00797	0.00534	0.00033	0.00056	0.00030	0.00101	0.16126	0.14741	0.14323	0.11028
ORG$_1$	red cabbage	S6	AV	0.00000	0.01037	0.01310	0.00238	0.00040	0.00000	0.00084	0.00180	0.02218	0.01775	0.15708	0.06625
			SD	0.00000	0.00508	0.00488	0.00412	0.00069	0.00000	0.00073	0.00090	0.00420	0.01119	0.03095	0.04976
IGZ	red cabbage	Redma RZ F1, 10.10.2019	AV	0.01913	0.27761	0.32086	0.04992	0.00257	0.00008	0.00827	0.00172	0.12183	0.05821	1.67900	0.54565
			SD	0.05412	0.02240	0.02491	0.01197	0.00032	0.00022	0.00296	0.00105	0.00670	0.01020	0.19672	0.07269

			precursor GLS	5MSOP	2PE		I3M										
			µmol/g fresh weight	5MSOP-ITC	2PE-CN	2PE-ITC	IAN	Sum ITC	% ITCs	Sum ETNs	% ETNs	Sum CN	% CNs	Sum Allyl	%AITC	%ACN	%CETP
CON$_1$	white cabba	S1	AV	0.00000	0.00095	0.00217	0.00141	0.305	52.78	0.184	31.98	0.088	15.24	0.195	30.62	7.18	62.20
			SD	0.00000	0.00020	0.00039	0.00042	0.048	7.02	0.027	5.24	0.017	2.31	0.019	2.80	0.24	2.56
CON$_1$	white cabba	S2	AV	0.00000	0.00107	0.00259	0.00118	0.219	36.17	0.274	44.37	0.121	19.47	0.246	18.16	7.09	74.76
			SD	0.00000	0.00063	0.00140	0.00091	0.020	5.26	0.064	6.15	0.038	3.44	0.063	2.21	1.91	0.73
CON$_1$	white cabba	S3	AV	0.00000	0.00029	0.00207	0.00011	0.170	46.54	0.115	29.97	0.095	23.49	0.155	30.04	4.64	65.32
			SD	0.00000	0.00050	0.00135	0.00020	0.034	7.99	0.044	1.96	0.050	6.87	0.034	15.03	4.24	10.81
CON$_1$	white cabba	S4	AV	0.00000	0.00000	0.00131	0.00379	0.474	40.93	0.265	22.99	0.414	36.08	0.348	20.08	5.95	73.96
			SD	0.00000	0.00000	0.00114	0.00546	0.103	6.74	0.050	3.49	0.030	4.25	0.025	12.93	0.75	13.67
CON$_1$	white cabba	S5	AV	0.00000	0.00000	0.00116	0.00062	0.497	42.50	0.320	27.89	0.341	29.61	0.472	27.60	4.91	67.48
			SD	0.00000	0.00000	0.00114	0.00017	0.193	13.02	0.058	5.61	0.088	7.56	0.060	10.10	0.40	10.08
CON$_1$	white cabba	S6	AV	0.00000	0.00000	0.00000	0.00610	0.150	20.51	0.181	24.47	0.407	55.02	0.129	22.32	0.00	77.68
			SD	0.00000	0.00000	0.00000	0.00317	0.005	2.02	0.031	0.94	0.063	1.12	0.019	2.26	0.00	2.26
CON$_2$	white cabba	S1	AV	0.00000	0.00064	0.00049	0.00191	0.230	38.47	0.145	25.26	0.208	36.27	0.167	17.70	11.69	70.61
			SD	0.00000	0.00008	0.00011	0.00048	0.101	12.52	0.024	5.48	0.019	7.50	0.028	5.98	1.72	4.26
CON$_2$	white cabba	S2	AV	0.00188	0.00027	0.00243	0.00015	0.529	63.06	0.155	18.29	0.160	18.65	0.228	34.46	4.88	60.66
			SD	0.00326	0.00024	0.00085	0.00025	0.076	11.36	0.054	5.96	0.068	6.42	0.036	14.36	0.85	13.51
CON$_2$	white cabba	S3	AV	0.00000	0.00000	0.00072	0.00054	0.358	54.63	0.105	19.40	0.137	25.97	0.188	37.25	6.57	56.18
			SD	0.00000	0.00000	0.00067	0.00071	0.318	13.29	0.044	5.43	0.050	7.91	0.134	15.30	2.21	13.72
CON$_2$	white cabba	S4	AV	0.00000	0.00000	0.00178	0.00084	0.382	39.83	0.266	27.98	0.306	32.19	0.370	25.88	4.72	69.40
			SD	0.00000	0.00000	0.00032	0.00146	0.075	5.57	0.016	3.17	0.026	3.45	0.012	5.26	0.35	5.45
CON$_2$	white cabba	S5	AV	0.00000	0.00000	0.00143	0.00157	0.148	27.88	0.238	46.17	0.136	25.95	0.265	19.85	5.51	74.64
			SD	0.00000	0.00000	0.00006	0.00271	0.059	9.14	0.029	9.94	0.025	2.73	0.012	9.34	1.20	8.63
CON$_2$	white cabba	S6	AV	0.00000	0.00000	0.00136	0.00205	0.320	39.41	0.292	34.82	0.221	25.77	0.350	30.05	5.85	64.10
			SD	0.00000	0.00000	0.00026	0.00131	0.023	8.80	0.069	2.33	0.093	6.74	0.052	7.05	1.24	7.33
ORG$_1$	white cabba	S1	AV	0.00000	0.00037	0.00412	0.00054	0.961	75.11	0.163	12.78	0.154	12.11	0.338	50.28	5.94	43.77
			SD	0.00000	0.00008	0.00135	0.00047	0.139	3.27	0.033	1.96	0.024	1.33	0.056	3.19	0.93	2.42
ORG$_1$	white cabba	S2	AV	0.00000	0.00012	0.00000	0.00348	0.591	43.55	0.185	38.04	0.251	18.42	0.238	17.42	7.34	75.25
			SD	0.00000	0.00021	0.00000	0.00260	0.622	32.82	0.054	41.13	0.302	9.83	0.056	19.18	1.63	18.41
ORG$_1$	white cabba	S3	AV	0.00000	0.00044	0.00000	0.00319	0.028	11.63	0.088	38.59	0.117	49.78	0.095	1.69	8.88	89.43
			SD	0.00000	0.00077	0.00000	0.00118	0.011	2.28	0.008	4.53	0.031	2.76	0.006	2.93	1.36	2.84
ORG$_1$	white cabba	S4	AV	0.00403	0.00000	0.00028	0.00338	0.496	53.10	0.157	15.89	0.293	31.01	0.209	29.22	7.35	63.44
			SD	0.00350	0.00000	0.00049	0.00040	0.136	13.01	0.090	5.34	0.121	9.49	0.094	9.32	0.92	8.47
ORG$_1$	white cabba	S5	AV	0.00276	0.00042	0.00062	0.00780	0.044	8.96	0.113	25.92	0.286	65.13	0.117	8.41	10.30	81.29
			SD	0.00477	0.00073	0.00054	0.00481	0.038	5.47	0.028	4.91	0.063	6.80	0.038	8.81	0.99	8.70
ORG$_1$	white cabba	S6	AV	0.00157	0.00027	0.00000	0.00532	0.022	6.67	0.096	29.43	0.209	63.90	0.104	9.97	5.70	84.33
			SD	0.00273	0.00046	0.00000	0.00267	0.007	0.57	0.035	0.80	0.074	1.36	0.031	1.95	4.97	3.73
IGZ	white cabba	Dottenfelder Dauer, 29.10.2019	AV	0.00000	0.00000	0.00000	0.02407	0.459	29.98	0.535	33.61	0.593	36.41				
			SD	0.00000	0.00000	0.00000	0.02848	0.132	8.82	0.196	6.78	0.251	12.50				
CON$_1$	red cabbage	S1	AV	0.02976	0.00172	0.00923	0.00000	1.730	75.37	0.299	13.49	0.251	11.13	0.121	48.31	0.00	51.69
			SD	0.00688	0.00069	0.00588	0.00000	0.366	7.13	0.091	5.47	0.021	1.67	0.036	11.89	0.00	11.89
CON$_1$	red cabbage	S2	AV	0.00964	0.00091	0.00219	0.00064	0.883	69.61	0.148	13.99	0.160	16.41	0.102	32.94	3.32	63.74
			SD	0.01670	0.00044	0.00267	0.00045	0.809	9.31	0.081	3.74	0.059	5.76	0.019	18.97	3.09	15.90
CON$_1$	red cabbage	S3	AV	0.00000	0.00074	0.00143	0.00013	0.424	52.24	0.231	20.61	0.217	27.15	0.106	30.67	1.09	68.25
			SD	0.00000	0.00128	0.00187	0.00023	0.309	16.76	0.299	12.05	0.172	9.79	0.067	8.39	1.88	8.53
CON$_1$	red cabbage	S4	AV	0.00000	0.00000	0.00089	0.00027	0.473	31.48	0.282	18.74	0.749	49.78	0.239	20.35	4.08	75.56
			SD	0.00000	0.00000	0.00091	0.00047	0.205	13.61	0.087	5.38	0.159	8.25	0.038	18.71	0.25	18.48
CON$_1$	red cabbage	S5	AV	0.00000	0.00328	0.00195	0.00224	0.200	7.42	1.194	45.45	1.291	47.13	0.335	4.09	3.71	92.20
			SD	0.00000	0.00057	0.00040	0.00031	0.060	1.55	0.176	8.98	0.439	7.89	0.095	0.36	0.20	0.39
CON$_1$	red cabbage	S6	AV	0.00000	0.00325	0.00026	0.00361	0.178	7.59	0.786	34.51	1.329	57.90	0.236	4.22	1.27	94.51
			SD	0.00000	0.00072	0.00045	0.00083	0.065	1.78	0.086	2.92	0.215	1.32	0.024	1.33	2.19	1.50
CON$_2$	red cabbage	S1	AV	0.00734	0.00152	0.00566	0.00013	0.681	51.86	0.332	23.81	0.334	24.32	0.227	31.49	6.20	62.31
			SD	0.00847	0.00053	0.00179	0.00022	0.056	12.73	0.147	7.72	0.113	5.03	0.019	8.84	1.28	9.99
CON$_2$	red cabbage	S2	AV	0.00100	0.00048	0.00060	0.00086	0.272	51.58	0.114	22.49	0.138	25.93	0.091	16.52	4.98	78.50
			SD	0.00173	0.00002	0.00019	0.00122	0.127	15.35	0.029	5.92	0.065	10.63	0.017	12.37	0.95	11.53
CON$_2$	red cabbage	S3	AV	0.00000	0.00112	0.00236	0.00000	0.562	44.07	0.353	29.96	0.303	25.97	0.137	27.59	1.30	71.11
			SD	0.00000	0.00097	0.00076	0.00000	0.345	21.96	0.112	11.71	0.088	11.51	0.041	18.65	2.25	20.48
CON$_2$	red cabbage	S4	AV	0.00000	0.00159	0.00113	0.00073	0.238	18.70	0.368	25.71	0.774	55.59	0.226	10.67	4.52	84.81
			SD	0.00000	0.00049	0.00115	0.00066	0.152	15.16	0.198	3.84	0.361	14.87	0.096	7.68	1.46	6.33
CON$_2$	red cabbage	S5	AV	0.00382	0.00301	0.00306	0.00124	0.347	19.45	0.857	39.33	0.798	41.21	0.338	22.70	2.36	74.94
			SD	0.00662	0.00264	0.00207	0.00110	0.169	8.85	0.554	13.05	0.369	4.37	0.151	19.40	2.05	17.39
CON$_2$	red cabbage	S6	AV	0.00613	0.00293	0.00114	0.00096	0.124	11.81	0.404	42.25	0.479	45.94	0.259	10.06	3.82	86.13
			SD	0.00531	0.00081	0.00100	0.00100	0.073	6.02	0.053	9.83	0.240	7.70	0.025	6.20	0.34	6.54
ORG$_1$	red cabbage	S1	AV	0.00000	0.00046	0.00285	0.00069	0.597	71.60	0.101	12.16	0.135	16.23	0.119	42.09	6.57	51.34
			SD	0.00000	0.00010	0.00142	0.00005	0.040	1.24	0.011	1.15	0.006	1.28	0.026	6.73	2.47	4.27
ORG$_1$	red cabbage	S2	AV	0.00000	0.00073	0.00181	0.00590	1.006	59.96	0.266	16.63	0.366	23.41	0.291	24.26	6.59	69.14
			SD	0.00000	0.00018	0.00121	0.00346	0.366	9.48	0.029	2.58	0.008	6.94	0.089	11.44	1.95	12.88
ORG$_1$	red cabbage	S3	AV	0.00000	0.00000	0.00000	0.00000	0.116	58.20	0.018	13.36	0.054	28.43	0.032	30.78	12.36	56.86
			SD	0.00000	0.00000	0.00000	0.00000	0.074	19.11	0.007	9.47	0.050	16.48	0.014	18.15	10.70	9.78
ORG$_1$	red cabbage	S4	AV	0.00000	0.00000	0.00169	0.00163	0.300	39.81	0.111	21.93	0.306	38.27	0.142	26.98	9.47	63.55
			SD	0.00000	0.00000	0.00053	0.00222	0.194	13.50	0.022	17.14	0.267	16.27	0.038	6.36	2.17	4.28
ORG$_1$	red cabbage	S5	AV	0.00232	0.00000	0.00000	0.00248	0.260	21.11	0.130	14.67	0.619	64.22	0.076	7.19	2.68	90.13
			SD	0.00401	0.00000	0.00000	0.00179	0.285	11.72	0.033	4.53	0.298	7.53	0.071	12.45	4.64	17.09
ORG$_1$	red cabbage	S6	AV	0.00000	0.00000	0.00037	0.00071	0.100	27.05	0.057	17.20	0.185	55.75	0.026	19.54	0.00	80.46
			SD	0.00000	0.00000	0.00032	0.00123	0.070	10.29	0.010	3.14	0.033	7.46	0.008	17.06	0.00	17.06
IGZ	red cabbage	Redma RZ F1, 10.10.2019	AV	0.00146	0.00436	0.00091	0.00412	0.696	19.57	1.017	28.42	1.865	52.01				
			SD	0.00412	0.00072	0.00119	0.00131	0.113	3.93	0.104	2.16	0.212	3.34				
