# Supplementary material for: Seasonal Variation of Glucosinolate Hydrolysis Products in Commercial White and Red Cabbages (Brassica oleracea var. capitata)
Source: Foods. 2020 Nov 17;9(11):1682. doi: 10.3390/foods9111682 (PMC7698573; doi:10.3390/foods9111682)
Supplement: Supplementary file 1 [file foods-09-01682-s001.zip › Table S4.pdf]

Table S4: Climatic data of the Dithmarschen region in northern Germany in 2019. Abbreviation: AV: average.

Elpersbüttel (Dithmarschen, Schleswig-Holstein), Germany

source: https://www.wetterkontor.de/de/wetter/deutschland/rueckblick.asp?id=52, accessed 05.11.2020

2019	AV T (min) [°C]	AV T (max) [°C]	AV T (average) [°C]	precipitation per month (L/m^2)	AV sunshine (h/d)	AV Wind (Bft)
May	6.2	15.0	11.0	39.1	6.7	6.0
June	11.8	23.0	17.7	66.3	8.9	5.6
July	14.0	21.5	17.6	43.3	5.9	5.8
August	13.5	23.3	18.5	51.9	7.3	5.6
September	10.7	18.2	14.5	117.7	4.8	6.0
October	7.4	13.8	10.6	92.9	3.1	5.7
November	3.4	8.1	5.9	64.8	1.3	5.2
